# Domain of Dentine Sialoprotein Mediates Proliferation and Differentiation of Human Periodontal Ligament Stem Cells

**DOI:** 10.1371/journal.pone.0081655

**Published:** 2013-12-03

**Authors:** Alkan Ozer, Guohua Yuan, Guobin Yang, Feng Wang, Wentong Li, Yuan Yang, Feng Guo, Qingping Gao, Lisa Shoff, Zhi Chen, Isabel C. Gay, Kevin J. Donly, Mary MacDougall, Shuo Chen

**Affiliations:** 1 Department of Developmental Dentistry, Dental School, The University of Texas Health Science Center at San Antonio, San Antonio, Texas, United States of America; 2 Department of Orthodontics, YüzüncüYıl University, Faculty of Dentistry, Kampus-Van, Turkey; 3 State Key Laboratory Breeding Base of Basic Science of Stomatology (Hubei-MOST) and Key Laboratory for Oral Biomedicine of Ministry of Education, School and Hospital of Stomatology, Wuhan University, Wuhan, China; 4 Department of Periodontics & Dental Hygiene, School of Dentistry, The University of Texas Health Science Center at Houston, Houston, Texas, United States of America; 5 Department of Oral/Maxillofacial Surgery, University of Alabama, Birmingham, Alabama, United States of America; Northwestern University, United States of America

## Abstract

Classic embryological studies have documented the inductive role of root dentin on adjacent periodontal ligament differentiation.  The biochemical composition of root dentin includes collagens and cleavage products of dentin sialophosphoprotein (DSPP), such as dentin sialoprotein (DSP).  The high abundance of DSP in root dentin prompted us to ask the question whether DSP or peptides derived thereof would serve as potent biological matrix components to induce periodontal progenitors to further differentiate into periodontal ligament cells. Here, we test the hypothesis that domain of DSP influences cell fate. In situ hybridization and immunohistochemical analyses showed that the COOH-terminal DSP domain is expressed in mouse periodontium at various stages of root development. The recombinant COOH-terminal DSP fragment (rC-DSP) enhanced attachment and migration of human periodontal ligament stem cells (PDLSC), human primary PDL cells without cell toxicity. rC-DSP induced PDLSC cell proliferation as well as differentiation and mineralization of PDLSC and PDL cells by formation of mineralized tissue and ALPase activity. Effect of rC-DSP on cell proliferation and differentiation was to promote gene expression of tooth/bone-relate markers, transcription factors and growth factors. The results for the first time showed that rC-DSP may be one of the components of cell niche for stimulating stem/progenitor cell proliferation and differentiation and a natural scaffold for periodontal regeneration application.

## Introduction

The dental attachment apparatus consists of two mineralized tissues; cementum and alveolar bone, with an interposed fibrous, cellular and vascular soft connective tissue termed the periodontal ligament (PDL). The PDL provides anchorage and support to the functional teeth and contributes to tooth nutrition, homoeostasis and repair of damaged periodontal tissue [1,2]. 

Periodontitis is an inflammatory disease that causes the destruction of periodontium including alveolar bone, gingiva, PDL and root cementum. Periodontal disease is the main cause of tooth loss and is a substantial public health burden worldwide [3,4]. The reconstruction of healthy periodontium destroyed by the periodontal diseases is a major goal of periodontal therapy. 

The PDL contains heterogeneous cell populations that are able to differentiate into cementum forming cells (cementoblasts) and bone-forming cells (osteoblasts) [1,5,6] and thus represents a potentially valuable source of clinical material for tissue repair and regeneration. Recently, stem cells in periodontal tissue have been isolated and characterized from various species. It includes gingival mesenchymal stem cells (gingival MSCs) [7-9], periodontal ligament stem cells (PDLSCs) [10-14], alveolar bone mesenchymal stem cells (alveolar bone MSCs) [15,16] and dental follicle progenitors/stem cells [17-19]. These progenitors/stem cells are capable of differentiating into bone, PDL and cement as well as provide the potential formation of true PDL apparatus in given environments *in vitro* and *in vivo*. Although the fine mechanisms regulating the proliferation and differentiation of mesenchymal precursors/stem cells into cementoblasts, osteoblasts and PDL cells during periodontal development and regeneration have not been wholly elucidated, it has been known that proliferation and differentiation of the progenitors/stem cells are controlled by various growth factors, transcriptional factors, host modulating agents and extracellular matrix (ECM) [20,21]. ECM not only mediates cell attachment and presents key cues to cell migration, but also it often provides specific microenvironments (niches) for cell growth and differentiation. ECM can be mimicked by synthetically tethering specific factors to substrates [22], which have been used to enhance survival of progenitor/stem cells [23] and to regulate selected transcriptional networks in these cells [24]. For instance, bone ECM influences osteoblast differentiation into osteocytes whereas dental ECM governs dental pulp stem cell differentiation into odontoblasts [25,26]. Therefore, niches influence cell behavior and fate [27,28].

Dentin sialoprotein (DSP) and dentin phosphoprotein (DPP) are cleaved products of dentin sialophosphoprotein (DSPP) [29]. The expression of DSP and DPP is found in bone and many normal tissues [30,31] besides teeth [32,33]. DSP and DPP have their unique biological roles during tooth development and formation [34-36]. Mutations of either DSP or DPP domain in the human cause various types of inherited dental disorders [30,36,37]. In DSPP null mice ^(-/-)^, abnormal bone and cementum structures along with the detachment of PDL were observed [38,39], implying that this protein is relevant to biological functions of periodontium. The full-length DSPP is hardly detected in extracts from osteoblasts and dental cells as well as bones and teeth [40-43]. This indicates that DSPP is catalyzed after translation. Our and other laboratories found that DSP is further processed into certain small fragments [41,44] whereas DPP is resistant to proteinases due to a unique composition with rich aspartic acid and phosphoserine residues comprising more than 85% of the extensive triple amino acid repeat motifs. DSP expression was detected in mouse periodontal tissue, implying an effect of the DSP on PDL biological functions [44,45].

In the present study, we investigated different expression patterns of DSP fragments in mouse periodontal tissue and found that the both NH_2_- and COOH-terminal DSP fragments are expressed in PDL cells. Expression of the COOH-terminal DSP domain, but no evidence of the NH_2_-terminal DSP domain expression, is seen in cement by immunohistochemistry analysis. The COOH-terminal DSP domain has a stimulatory effect on cell attachment, migration, proliferation and differentiation of human PDLSC and primary PDL cells *in vitro*. Furthermore, we identified gene expression that facilitates cell proliferation and differentiation.

##  Materials and Methods

### Animal and tissue preparation

The protocol of animal use was approved by the Animal Welfare Committee at the University of Texas Health Science Center at San Antonio (UTHSCSA). C57BL/6 mice were scarified at the ages of postnatal day 10 (PD10), 14 and 1.5, 2.0, 7.5 and 13.5 months (M). At least three mice for each time point were taken for the following analyses. Mandibles of the mice were immediately dissected and immersed into 4% paraformaldehyde in 0.1M phosphate buffer (pH 7.4) for 2 days at 4°C. After washes with phosphate buffer saline (PBS), samples were demineralized in 8% EDTA for 2-8 weeks. Then the tissues were processed for paraffin embedding and 5-μm sagittal serial sections were prepared.

### Cell isolation and cell culture

Isolation of human primary periodontal ligament (PDL), periodontal ligament stem cells (PDLSCs) and primary gingival fibroblast (GF) cells was described previously [11,46]. Briefly, disease-free impacted third molars were collected from ten individuals aged 18–26 years old at the Oral and Maxillofacial Surgery Department at UTHSCSA. The institutional review board at the UTHSCSA approved this study and informed consent was obtained from all participants. The periodontal ligament and gingival tissues were scraped and kept separately according to their anatomical locations. These cells were digested for 1 h at 37°C in a solution of 3 mg⁄ml collagenase type I and 4 mg⁄ml of dispase (Worthington Biochem, Freehold, NJ). Primary cells from donors were pooled together at the same passage to obtain a primary cell type, either PDL or GF cells, used for fluorescence-activated cell sorting (FACS) and expanded for additional experiments. For FACS, approximately 2 x 10^6^ cells per sample of each type were used. The samples were labeled using a mixture of 100 µl of primary antibody (STRO-1 antibody: Life Technologies, Grand Island; NY), 50 µl of normal goat serum (Life Technologies) and 350 µl of FACS wash buffer (3% FBS and 0.25 M EDTA in PBS). Cells were then washed three times with 10 ml of FACS wash buffer, labeled using 5 µl secondary antibody (Alexa Fluor 488, Life Technologies) and sorted. The negative control lacked primary antibody was stored on ice for 30 min in 3 µl secondary antibody (Alexa Fluor 488, Life Technologies). The second negative control consisted of approximately 3 x 10^5^ cells stored in 300 µl of FACS wash buffer only, and sorted [46]. Isolation of STRO-1-positive cells was performed on a FACS AriaII from BD Biosciences (San Jose, CA) and data were analyzed using BD FACS DIVA software at the Flow Cytometry and Cellular Imaging Core Facility (MD Anderson Cancer Center, Houston, TX). Enriched STRO-1 PDLSC cells were obtained, cultured and expanded. Other stem cell markers were used to further confirm the PDLSC cells including CD 105, CD 29 (Integrin β1), stage-specific embryonic antigen-4 (SSEA4) and CDT4 (ab44967, ab30394, ab16287 and ab27985, respectively; Abcam, Boston MA, 1:25, 1:25, 1:10 and 1:50 dilution). As a negative control, a mouse IgG1 isotype monoclonal antibody (ab27479; Abcam) was used. The PDLSC cells were expanded in Dulbecco’s modified Eagle’s medium (DMEM) containing 10% FCS and 1% antibiotics at 37°C in a 5% CO_2_ atmosphere. The primary PDL and GF cells with negative stem cell markers were grown under the same conditions as the PDLSC cells. All of the experiments were performed in triplicate from three independent experiments using the same batch of tissues of all combined donors.

### In situ hybridization

Mouse mandibles were fixed and processed. Serial sections prepared in either the sagittal or frontal plane were mounted on saline-treated slides. Representative sections from each block were stained with hematoxylin. A 479-bp mouse cDNA corresponding to a coding region of the DSP segment of DSPP gene was generated by PCR [32]. Amplified PCR product was subcloned into pCRII vector containing Sp6 and T7 promoters (Invitrogen, Carlsbad, CA). Labeled ^32^P-rUTP antisense and sense DSP probes were generated using Sp6 or T7 RNA polymerases after linearization with appropriate restriction enzymes. The method of *in situ* hybridization was performed as described earlier [47]. Briefly, hybridization was performed at 55°C overnight in a solution containing 50% formamide, 20 mM Tris-HCl (pH 8.0), 1 mM EDTA, 0.3 M NaCl, 10% dextran sulfate, 1 x Denhardt’s solution, 100 µg/ml denatured SS-DNA, 500 units/ml tRNA, and 1 µg/ml of ^32^P-rUTP labeled RNA probe. After hybridization, the cover slips were removed in 2 x SSC at room temperature, and sections were washed in RNase-free buffer (0.3 M NaCl, 10 mM Tris-HCl, 5 mM EDTA) at 37°C for 10 min. The sections were incubated with RNases (40 mg/ml RNase A1 and 10 U/ml RNase T1) in the RNase-free buffer at 37°C for 1 h, followed by incubation in the RNase-free buffer for 30 min. Consecutive 5-minute washes at 57°C were done twice with 2 x SSC, four times in 0.5 x SSC, and three times in 0.1 x SSC. After washing, the sections were dehydrated using ethanol containing 0.3 M ammonium acetate. For autoradiography, slides were dipped in photographic emulsion (NTB 3; Kodak Scientific Imaging, Rochester, NY) diluted 1:1 with 0.6 M ammonium acetate at 42°C. After drying at room temperature, the slides were exposed in the presence of desiccant for 3 days to 3 weeks and developed in a Kodak D-19 developer. The slides were counter-stained with hematoxylin, dehydrated through ethanol, cleared in xylene, and mounted with Permount (SOP-1.5; Fisher Scientific, Pittsburgh, PA).

### Immunohistochemistry

Immunohistochemistry assay was performed with the use of the ABC Vectastain kit (Vector Laboratories, Inc., Burlingame, CA) according to the manual’s instruction. Paraffin-embedded tissue sections were incubated in a dry oven at 62°C for 1 h, and de-waxed slides were deparaffinized in xylene, hydrated with graded ethanol and incubated with hyaluronidase for 1 h at 37°C to expose the epitopes of target proteins. Then, the tissue samples were treated with 0.3% H_2_O_2_ in methanol solution to inactivate endogenous peroxidase. Afterwards, they were processed with a blocking buffer for 1 h at room temperature to block non-specific binding. For the detection of mouse DSP, a rabbit polyclonal anti-mouse DSP antibody recognizes residues between Ile^18^ and Lys^371^ as anti-NH_2_ terminal DSP antibody (M300; Santa Cruz Biotechnology Inc., Santa Cruz, CA) and another polyclonal anti-COOH terminal mouse DSP antibody was produced in rabbit using the oligopeptide with the sequence of KRNSPKQGESDKPQGTAE (mouse DSP residues 401-418, Alpha Diagnostic International, San Antonio, TX). Primary antibodies diluted in PBS (1:200 for anti-NH_2_ DSP antibody; 1:50 for anti-COOH DSP antibody) were applied for 16 h at 4°C. Those samples incubated with control Ig G instead of first antibody were used as negative control. Then, secondary antibodies were placed on the samples for 1 h at room temperature. After washes, they were treated with ABC solution (Vector Laboratories, Inc.) for 30 min at room temperature and immune-positive loci were detected by incubation with 3, 3’-diaminobenzidine tetra hydrochloride. Finally, the samples were counterstained with hematoxylin solution.

### Expression and purification of a recombinant COOH-terminal mouse dentine sialoprotein

The expression and purification of a recombinant COOH-terminal mouse DSP were performed according to previously described protocols [48]. The COOH-terminal domain of mouse DSP at residues between 183 and 457 was amplified by PCR using a full-length mouse DSPP cDNA as a temple with primers adding EcoRI sites at both ends for directional ligation into the expression vector pGEX-6P3 with EcoRI sites (Amersham Biosciences, Piscataway, NJ) and named rC-DSP. After confirming the right sequence, the resulting plasmid was transformed into Escherichia coli BL21. The rC-DSP expression and purification were performed according to the manufacturer’s instruction (Amersham Biosciences). Briefly, the rC-DSP protein was induced by the addition of isopropyl b-D-thiogalacto pyranoside (IPTG) and then purified using a GSTrap 4B purification system (Amersham Biosciences). The purified recombinant protein was analyzed by sodium dodecyl sulfate–polyacrylamide gel electrophoresis (SDS–PAGE) followed by Coomassie brilliant blue (CBB) staining and Western blotting analysis.

### Scanning electron microscopy (SEM)

The rC-DSP and collagen type I (Sigma-Aldrich, St. Louis, MO) in 0.1% (v/v) acetic acid were coated by spreading on coverslips (Fisher Scientific). Following protein coating, the coverslips were allowed to incubate at 37°C overnight. Surfaces were then allowed to dry thoroughly for 24 hours. Coverslips were washed in 0.1M sodium cacodylate buffer and fixed for 20 min in 2.5% (w/v) glutaraldehyde (Sigma-Aldrich) in 0.1M cacodylate buffer. The coverslips were washed in sodium cacodylate buffer and dehydrated in an ascending alcohol series. Finally, the coverslips were fixed in hexamethydioslazane (Sigma-Aldrich) allowed to air dry and then spuuter-coated with gold. The samples were examined by scanning electron microscopy (SEM) at kV (JEOLJSM 6610 LV; JEOL, Inc., Peabody, Mass).

### Cell damage assay

The cytotoxic effect of rC-DSP on cell damage was measured the release of lactate dehydrogenase (LDH) from damaged and dying cells using the CytoTox-96^®^ Non-Radioactive Cytotoxicity assay (Promega, Madison, WI). In brief, the cells were grown at DMEM with 10% FCS and 1% antibiotics and treated with rC-DSP at 5, 25 and 50 mM. At 1- , 3-, and 7-day treatments, LDH released from the cells into the culture medium was collected and quantified based on the enzymatic conversion of the tetrazolium salt into a red formazan product. The amount of LDH in medium was measured at 490 nm spectrophotometer according to the manufacturer’s instructions (Bio-Rad Laboratories, Inc. Hercules, CA). The cells without rC-DSP treatment as negative control were cultured at DMEM with 10% FCS and 1% antibiotics. 

### Cell attachment analysis

For cell attachment assay, 12-well tissue culture plates (TCPs) (Corning, Horseheads, NY) were firstly coated with 1, 5 and 10 mM of rC-DSP. The rC-DSP coated plates were incubated at 37°C overnight and then the plates were washed with 1 x cold PBS. PDLSC, PDL and GF cells (2 x 10^4^ cells/per well) were seeded into the plates and incubated for 120 min. Then, the cells were gently washed with PBS and removed unattached cells. The attached cells were counted under microscopy. On the other hand, the adherent cells were continued to grow at 37°C for overnight and then fixed for 20 min with 70% ethanol and washed twice in PBS. Fifty µl of a 5 mg/ml solution of crystal violet (Sigma-Aldrich) was added to each well for 10 min, and wells then washed with water for several times until no further dye was released. Fifty µl of citric acid (0.1 M, pH 4.2) was added to each well for 30 min with shaking to release the dye and then absorbance was measured at 550 nm on an absorbance spectrometer (MRX II, Dynex Technologies, Chantilly, VA). The rC-DSP coated onto the TCP was not stained by the solution of crystal violet. The cells were grown in TCPs only as control. 

### Cell migration assay

Human PDLSC, PDL and GF cells were maintained in DMEM medium supplemented with 10% FCS, 100 units/mL penicillin and 100 µg/mL streptomycin. Cell migration assay was used BD Falcon cell culture inserts incorporating polyethylene terephthalate (PET) track-etched membranes with 8 µM perforations (BD Biosciences). The cell culture inserts were placed in 12-well plates. Cells (5 x 10^4^ cells/ml) were added to the upper chamber in 250 µL DMEM containing 0.1% FCS. The lower chamber contained DMEM with or without 5, 25 and 50 mM of rC-DSP in 0.1% FCS as chemoattractant. After 12-hour incubation of 5% CO_2_ at 37°C, the chambers were disassembled, and the filters were fixed in methanol and stained with HemaDiff eosin and thiazine (Statlab, Lewisville, TX). The number of cells that had migrated through the filters was quantified by counting 10 fields per membrane at a 200-fold magnification. Experiments were performed in triplicate of three separate studies.

### Cell proliferation

Cell proliferation assay was performed by direct cell counting and MTT methods. Briefly, cells treated with and without 5, 25 and 50 mM of rC-DSP were seeded into 6-well plates at 5 x 10^4^ cells per well. The cells were trypsinized and counted using a hemocytometer for up to 15 days. For MTT assay, cells were seeded into 96-well plates with 1.5 x 10^3^ cells per well and detected at days 1, 3, 6, 9, 12 and 15, respectively, using MTT cell proliferation assay kit (ATCC, No. 30-1010K, Manassas, VA).

### Alkaline phosphatase (ALP) and mineralization assays

Cells were treated with or without 50 mM of rC-DSP in 6-well plates at a density of 4 x 10^5^ per well and cultured in calcifying medium (α-MEM supplemented with 10% FCS, penicillin (100 U/ml) and streptomycin (100 µg/ml), 50 µg/ml ascorbic acid and 10 mM sodium beta-glycerophosphate) at 37°C for 1 and 3 weeks, respectively. For detection of ALP activity, the cells were fixed with 70% ethanol for 5 min and washed in the buffer (100 mM Tris–HCl, pH 9.5; 100 mM NaCl; 50 mM MgCl_2_). In situ ALP staining was performed according to the supplier’s instructions (Bio-Rad Laboratories, Inc.). For quantitative ALP activity, the cell lysates was assayed using *p*-nitrophenyl-phosphate as a substrate. Protein concentration was determined using the bicinchoninic (BCA) protein assay reagent (Pierce, Rockford, IL). The enzyme activity was expressed as nanomoles of *p*-nitrophenol produced per min per mg of protein. For mineralization assays, the cells were fixed in 10% formaldehyde neutral buffer solution and then stained with Alizarin Red S (Sigma-Aldrich). Calcium content was measured according to the manufacturer’s instructions using Osteogenesis Assay kit (Millipore, Temecula, CA). Briefly, 400 µl of 10% acetic acid was added to each well and incubated for 30 min. Cells were scraped and transferred to microcentrifuge tubes. The tubes were heated to 85°C for 10 min and then transferred to ice for 5 min, followed by centrifugation. The supernatants were transferred to new microcentrifuge tubes and neutralized with 10% ammonium hydroxide. Calcium content in the supernatants was determined by the Osteogenesis Assay kit and protein concentration was measured using the micro BCA Assay Reagent kit (Pierce). The experiments were performed in triplicate of three independent times.

### Quantitative real time PCR

Cells were treated with or without 50 mM of rC-DSP in DMEM containing 2% FSC and 1% antibiotics at 3, 5, 7 and 10 days, respectively. After treatment, total RNA was isolated using TRIZOL reagent (Qiagen Inc. Valencia, CA), treated with DNase I (Promega), and purified with the RNeasy Mini kit (Qiagen Inc.). RNA concentration was determined by UV spectroscopy at 260 nm. Complementary DNA synthesis and PCR amplification were performed using standard protocols. For quantitative real time PCR (qRT-PCR), amplification reaction was analyzed in real time on an ABI 7500 (Applied Biosystems, Foster City, CA) using SYBR Green chemistry, and threshold values were calculated using SDS2 software (Applied Biosystems). Primers used for qRT-PCR were shown in Tables S1 and S2. The ^ΔΔ^Ct method was used to calculate gene expression levels normalized to cyclophilin A values. The results were performed from triplicate of three separate experiments.

### Western blot analysis

Cells were treated with or without 50 mM of rC-DSP in DMEM medium with 2% FCS and 1% antibiotics at 37°C in a 5% CO_2_ atmosphere for 7 days. The cells were then washed with 1 x cold PBS and lysed with RIPA buffer (1 x PBS, 1% Nonidet P-40, 0.5% sodium deoxycholate, 0.1% SDS, 10 mg/ml phenylmethylsulfonyl fluoride, 30 µl/ml aprotinin, 100 mM sodium orthovanadate; Santa Cruz Biotechnology, Inc.). Whole cell lysates were resolved by 7 % SDS-PAGE gels and transferred to Trans-Blot membranes (Bio-Rad Laboratory, Inc.). For the detection of mouse DSP, the NH_2_- and COOH-terminal DSP antibodies were used. Rabbit polyclonal anti-human DMP1 (a gift from Dr. Larry Fisher, National Institute of Health, Bethesda, MD), rabbit polyclonal anti-human DLX3 and rabbit polyclonal anti-human OSX (Abcam), rabbit polyclonal anti-human osteocalcin (OC) antibody, goat polyclonal anti-human osteoprotegerin (OPG) antibody and goat polyclonal anti-human RUNX2 antibody (Santa Cruz Biotechnology, Inc.) were used as primary antibodies. The membranes were blocked with 5% non-fat milk in TBST buffer (10 mM Tris-HCl, pH 7.5, 100 mM NaCl, 0.1% Tween-20) for 60 min at room temperature. After washing, the membranes were incubated with primary antibodies against DMP1, DLX3, OC, OPG, OSX and RUNX2 with appropriate dilution (1:500-1,000) for overnight at 4°C, respectively. The secondary antibody (horseradish peroxidase-conjugated anti-rabbit or anti-goat IgG) were used a dilution of 1:5,000-10,000 at room temperature for 60 min. Immunoreactivity was determined using ECL chemiluminescence reagent (Amersham Biosciences). As a control, goat polyclonal anti-human β-actin antibody was used (Santa Cruz Biotechnology, Inc.). For detection of DSP expression patterns, mouse pre-odontoblast-like (MD10-F2) cells were grown in α-MEM medium supplemented with 10% FCS, 100 units/ml penicillin, 100 µg/mL streptomycin, 50 µg/ml ascorbic acid and 10 mM β-glycerphosphate at 33°C under 5% CO_2_. The cells were washed with 1 x cold PBS and lysed with a RIPA buffer. The whole cell lysates were resolved by a 7% SDS-PAGE gel and transferred to a Trans-Blot membrane (Bio-Rad Laboratory, Inc.). Western blot assay was performed as described above using the anti-NH_2_-DSP and anti-COOH-DSP antibodies, respectively.

### Statistical analysis

Quantitative data were presented as means ± S.D. from three independent experiments and compared with the results of one-way ANOVA using Statview software (SAS Institute Inc., Lary, NC). The differences between groups were statistically significant at *p < 0.05 and **p< 0.01.

## Results

### Expression of the COOH-terminal domain of DSP in mouse periodontium

**Figure 1:**
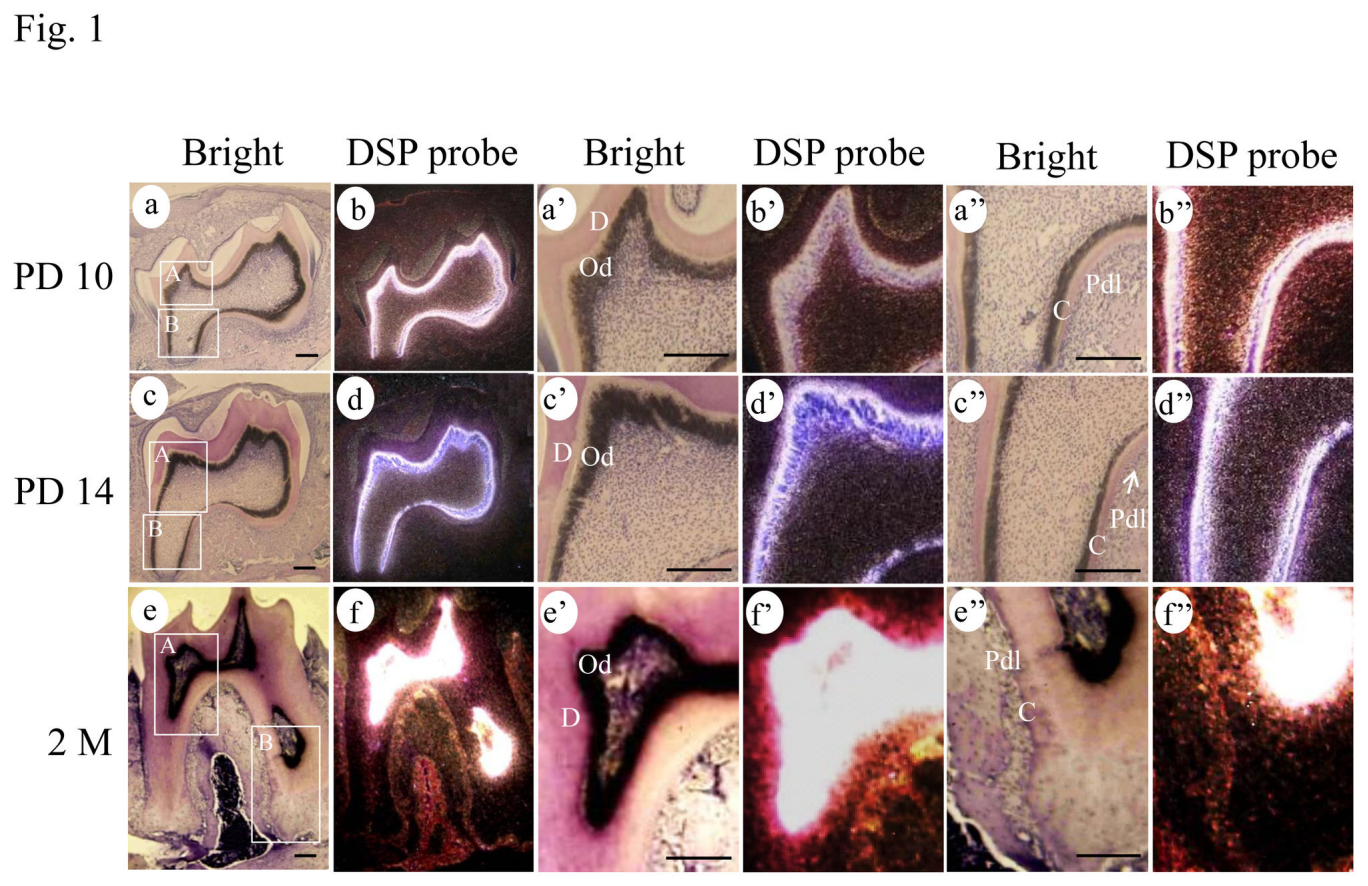
DSPP expression in mouse periodontium. In situ hybridization of DSPP in mouse teeth at postnatal days 10 (PD10), 14 and 2 months (2M). **a**-**f**. At PD10, PD14 and 2M, DSPP mRNA expression was apparently observed in odontoblasts and moderately in periodontal ligament cells. **a’-f**’ **and** **a**’’**-f**’’. Higher magnification of A and B boxes in a-f, showing the expression of DSPP mRNA in odontoblasts and periodontal ligament cells. C, cement; D, dentin; Od, odontoblasts; Pdl, periodontal ligament cells. a-f, bars 200 μM; a’-f’, bars 50 μM.

**Figure 2:**
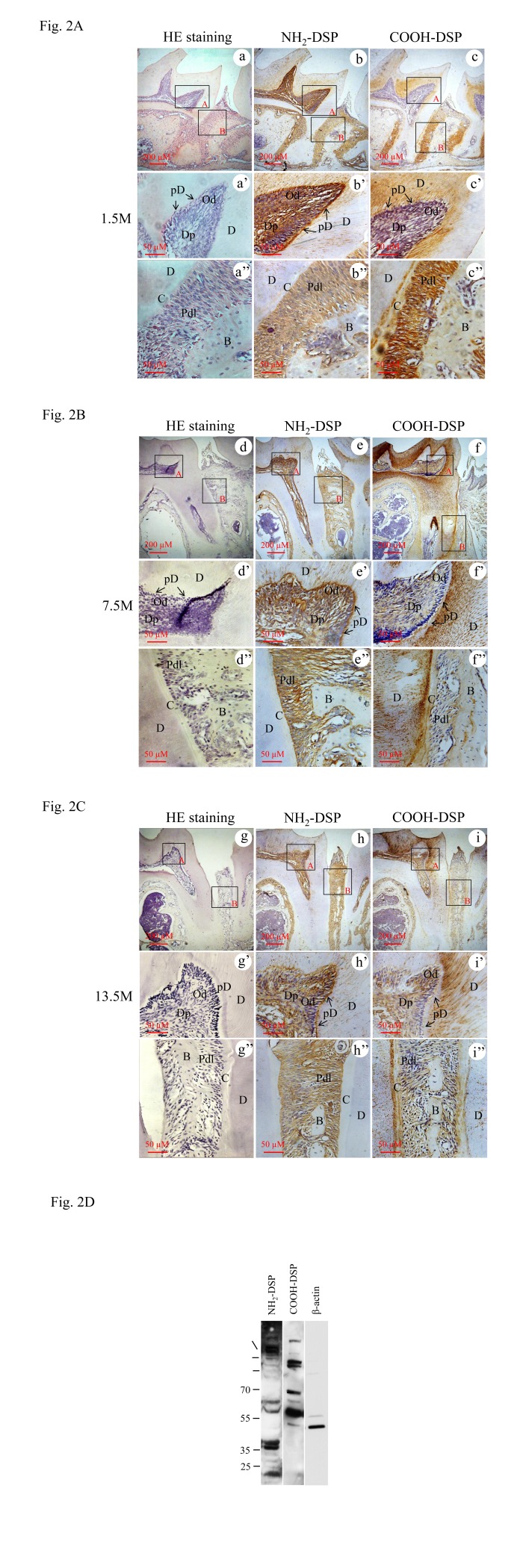
Immunolocalization and distribution of the NH_2_- and COOH-terminal DSP fragments in mouse teeth. **A**-**C**. Anti-NH_2_- and anti-COOH-terminal DSP domain antibodies in mouse first molars at 1.5M (b–c), 7.5M (e–f), 13.5M (h-i). HE staining of the first mandibular molars was shown at a, d and g. **b**, **e** and **h**. Intense immune distribution of the anti-NH_2-_terminal DSP was apparently detected in predentin, odontoblasts and dental pulp. Also, the NH_2_-terminal DSP domain was expressed in osteoblasts, PDL cells, but barely seen in cement at 1.5M (b), 7.5M (e) and 13.5 M (h). **b’-b’’**, **e’-e’’**, and **h’-h**’’ show higher magnification of A and B boxes in b, e and h. **c**, **f** and **i**. Anti-COOH DSP antibody showed strong reactions in dentinal tubules, but weakly in predentin compared to that of the anti-NH_2_-terminal DSP antibody. Expression of the COOH-terminal DSP domain was substantially seen in osteoblasts, PDL cells, and cement at 1.5M (c), 7.5M (f), and 13.5M (i). **c’-c’’** **, f’-f’’** **and** **i’-i’’**. Higher magnification of A and B boxes in c, f and i. **a**, **d** and **g**. Control tissue sections were incubated with normal IgG instead of first antibodies, showing a negative reaction. **a’-a’’**, **d’-d’’** and **g’-g’’** show higher magnification of A and B boxes in a, d and g. **D.** Western blot analysis of DSP expression patterns in mouse MD10-F2 cells using the NH_2_- and COOH-terminal DSP antibodies. Multiple NH_2_- and COOH-terminal DSP fragments were detected in MD10-F2 cells. β-actin was used as an internal control. B, bone; C, cement; D, dentin; Dp, dental pulp; pD, predentin; Pdl, periodontal ligament cells.

To determine DSPP expression in postnatal teeth, we performed *in situ* hybridization assay. The results showed that DSPP mRNA is strongly expressed in odontoblasts. However, its expression was also seen in alveolar osteoblasts and PDL cells at the postnatal days 10, 14 and two-month old mouse teeth (Figure 1). To further identify expressional profiles of DSP fragments in mouse periodontal tissue, immunohistochemistry was conducted using the NH_2_- and COOH-terminal DSP antibodies. The results showed that the NH_2_-terminal DSP domain was apparently detected in predentin, odontoblasts and dental pulp cells beneath the cusps, its expression was also substantially seen in mouse gingival fibroblasts, osteoblasts and PDL cells at the first molars of 1.5-month-old mice, but expression of the NH_2_-terminal DSP was barely detected in cementum. However, expression of the COOH-terminal DSP fragment was intensely observed not only in PDL cells, but also in cementum besides its expression was highly seen in the mineralized dentin and weakly in predentin, odontoblasts and dental pulp cells (Figure 2A). Similar to 1.5-month-old teeth, the distribution of the NH_2_- and COOH-terminal DSP fragments exhibited the same patterns at 7.5- and 13.5-month-old teeth (Figure 2B-C). To further identify different expression patterns of the NH_2_- and COOH-terminal DSP fragments, Western blot assay with whole cell lysates from mouse pre-odontoblast-like (MD10-F2) cells was performed using the NH_2_- and COOH-terminal DSP antibodies. The results showed that multiple bands were recognized by both the NH_2_- and COOH-terminal DSP antibodies, respectively (Figure 2D). To exclude the possibility that proteins from the pre-odontoblast-like cells were degraded during protein isolated process, β-actin was used as an internal control and a band at approximately 42 kDa was identified by Western blot assay (Figure 2D). These data indicate that DSP is processed into the NH_2_- and COOH-terminal domains in nature. 

### Matrix preparation

**Figure 3:**
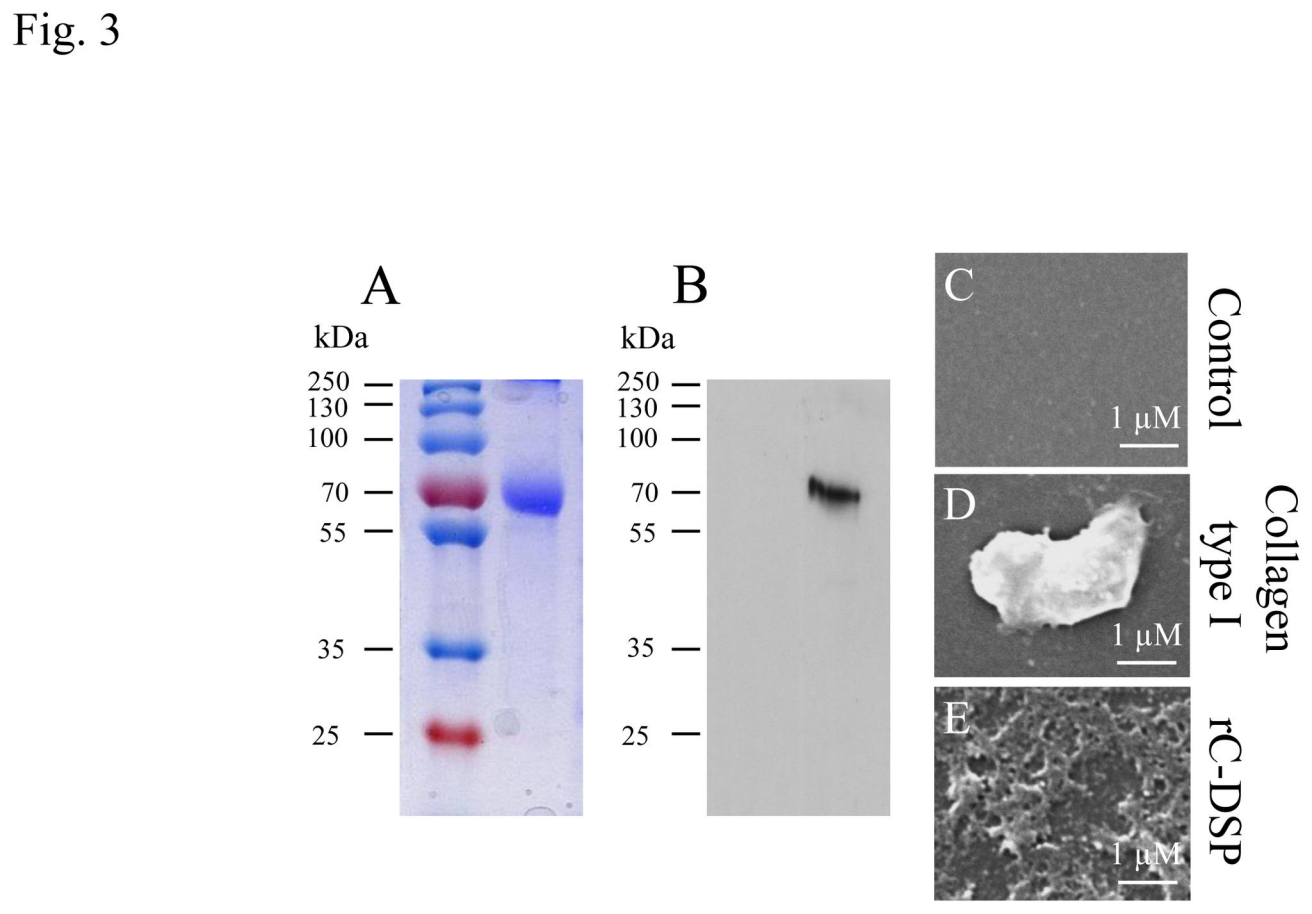
Product of DSP protein coated on glass slides. **A**. Coomassie-stained SDS-PAGE gel for analysis of expression of the recombinant COOH-terminal dentin sialoprotein (rC-DSP) protein. The sample in DL21 cells was grown 3 h with 1 mM IPTG induction after reaching O.D.600 of 0.6. The rC-DSP was purified by the column described in the materials and methods. **B**. Expression of purified rC-DSP was confirmed by Western blotting analysis using goat polyclonal anti-DSP antibody (Santa Cruz Biotechnology Inc.). **D**-**E**. Collagen type I and rC-DSP proteins were coated on glass slides for overnight at 37°C. For scanning electron microscopy, the samples were washed and dried as well as treated with sodium deoxycholate. Then the samples were observed under scanning electron microscope (SEM). Fibril matrix was visible on the collagen type I coated slide (**D**) and rC-DSP coated slide (**E**), but no control slide (**C**). Also, there were different morphologies between rC-DSP and collagen type I.

We found that the COOH-terminal DSP domain, as a ligand, interacts with cellular membrane protein, integrin β6, activating phosphorylation of Smad^1/5/8^ and p38 proteins (unpublished data). To assess the role of the COOH-terminal DSP fragment in PDL functions, a recombinant COOH-terminal DSP protein (rC-DSP) was generated and purified. The purified rC-DSP was resolved to near homogeneity as analyzed by a SDS-PAGE gel (Figure 3A) and further verified by Western blot assay using the anti COOH-terminal DSP antibody (Figure 3B). Then, we coated the purified rC-DSP and collagen type I onto glass slides and compared their morphologies. Under low magnification using phase contrast microscope, the protein matrices of rC-DSP and collagen type I were invisible (data not shown), but the protein matrix deposits of rC-DSP and collagen type I were visible at high magnification using SEM and displayed different morphologies (Figure 3D-E). In contrast, the control slide without protein matrix did not have such deposits when image at the same magnification (Figure 3C). 

### Cell cytotoxicity, attachment and migration assays

**Figure 4:**
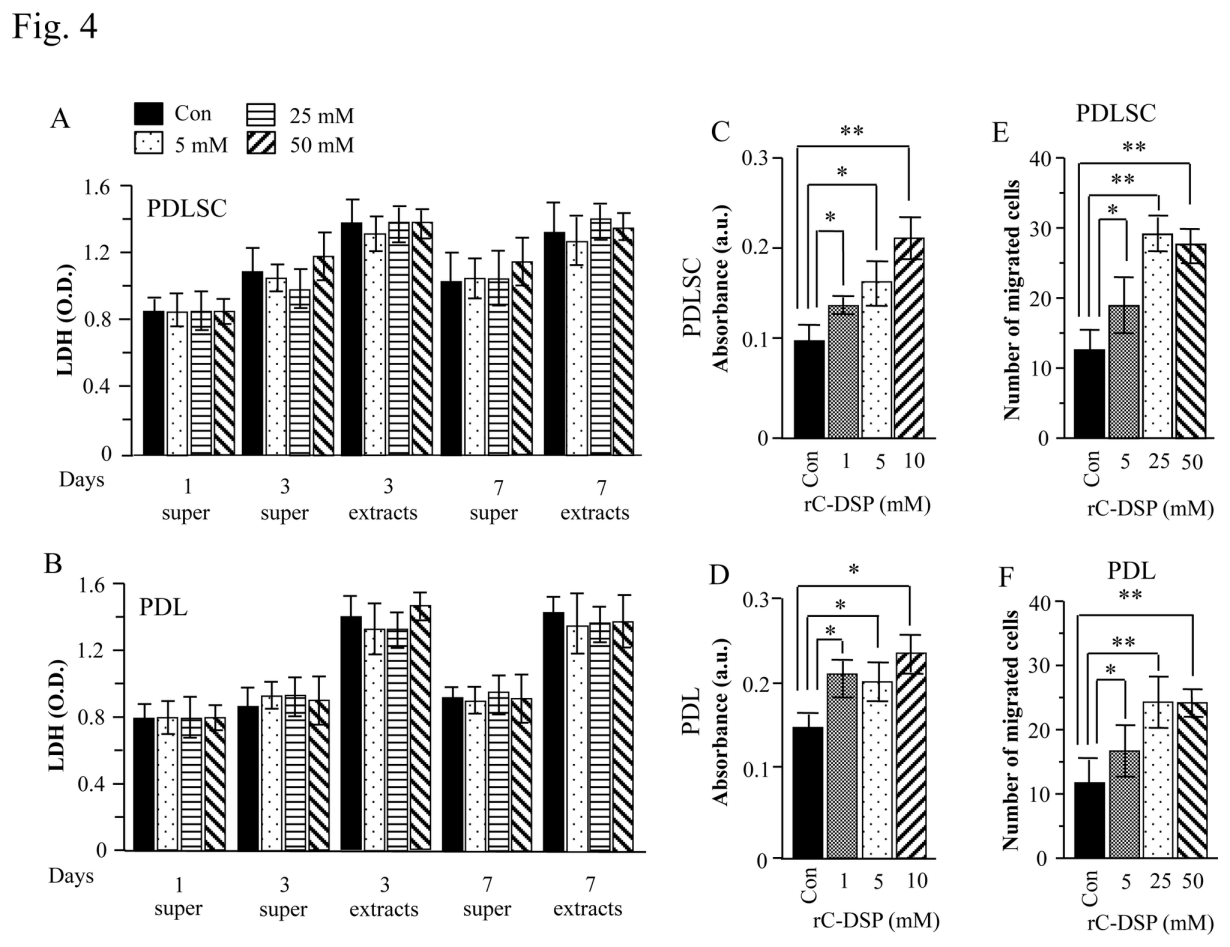
Effect of rC-DSP on cell damage, attachment and migration. **A**-**B**. The PDLSC and PDL cells were treated with the given doses of rC-DSP for 1, 3 and 7 days. LDH from the cell supernatant and extracts was quantitated using CytoTox 96^®^ non-radioactive cytotoxicity assay. Data are shown as mean percentage of damage/dead cells obtained by analysis of three separate experiments. Bar graphs represent means ± S.D. (n=3). There were no significant differences among control and rC-DSP treated groups. Super, cell supernatant. **C**-**D**. Cells were grown on coated with or without different concentrations of rC-DSP plates. After 12 h, adherent cells were fixed and washed with PBS. Crystal violet was then added to the cells and the absorbance of crystal violet taken by cells was measured at 550 nm on an absorbance spectrometer. rC-DSP coated onto the CTP was not stained by crystal violet. The results showed that rC-DSP induces PDLSC and PDL cell attachments. *p<0.05, **p<0.01. These tests were performed in triplicate from three independent experiments. **E**-**F**. Cells were treated with or without 5, 25 and 50 mM of rC-DSP for 16 h. Photographs represent the migrated cells. Concentration-dependent effect of rC-DSP was observed in PDLSC and PDL cells. Bar represents the mean ± S.D. (n = 3). *p< 0.05 vs control, **p< 0.01 vs control.

We firstly studied whether rC-DSP has any toxic to these cells. The PDLSC and PDL cells were presence of 5, 25 and 50 mM of rC-DSP for 1, 3 and 7 days. The cell media were then collected and LDH activity released from damaged cells was measured. At 1- , 3- and 7-day rC-DSP treatments, there were no significant differences of LDH activity between rC-DSP stimulated and unstimulated groups (Figure 4A-B). Intracellular LDH levels also showed no differences among these groups. Furthermore, rC-DSP had no toxic effects on GF cells (Figure S1A). Based on these experiments, we conclude that rC-DSP is a safe biomaterial. 

We then evaluated the effects of rC-DSP on attachment of human PDLSC and PDL cells. Cell attachment in the presence of different concentrations of rC-DSP coated onto plates was greater than the control groups at a 3-hour incubation (*p*<0.05) (data not shown). At a12-hour incubation, cell attachment was measured using the absorbance of crystal violet taken up by cells. The maximal effect of cell attachment was 10 mM of rC-DSP, and there were no significant differences with increasing rC-DSP concentrations (data not shown). The results showed that rC-DSP had a significant effect on these cell attachment (*p*<0.05) (Figure 4C-D). Also, rC-DSP promoted GF attachment (Figure S1B).

We next investigated whether the matrix has a stimulatory effect on cell migration. When different concentrations of rC-DSP were presence in the culture medium, the cell migration was increased compared to the unstimulated cells at overnight incubation (*p*<0.05) (Figure 4E-F, Figure S1C). These results indicate that rC-DSP promotes those cell attachment and migration. 

### Cell proliferation and differentiation

**Figure 5:**
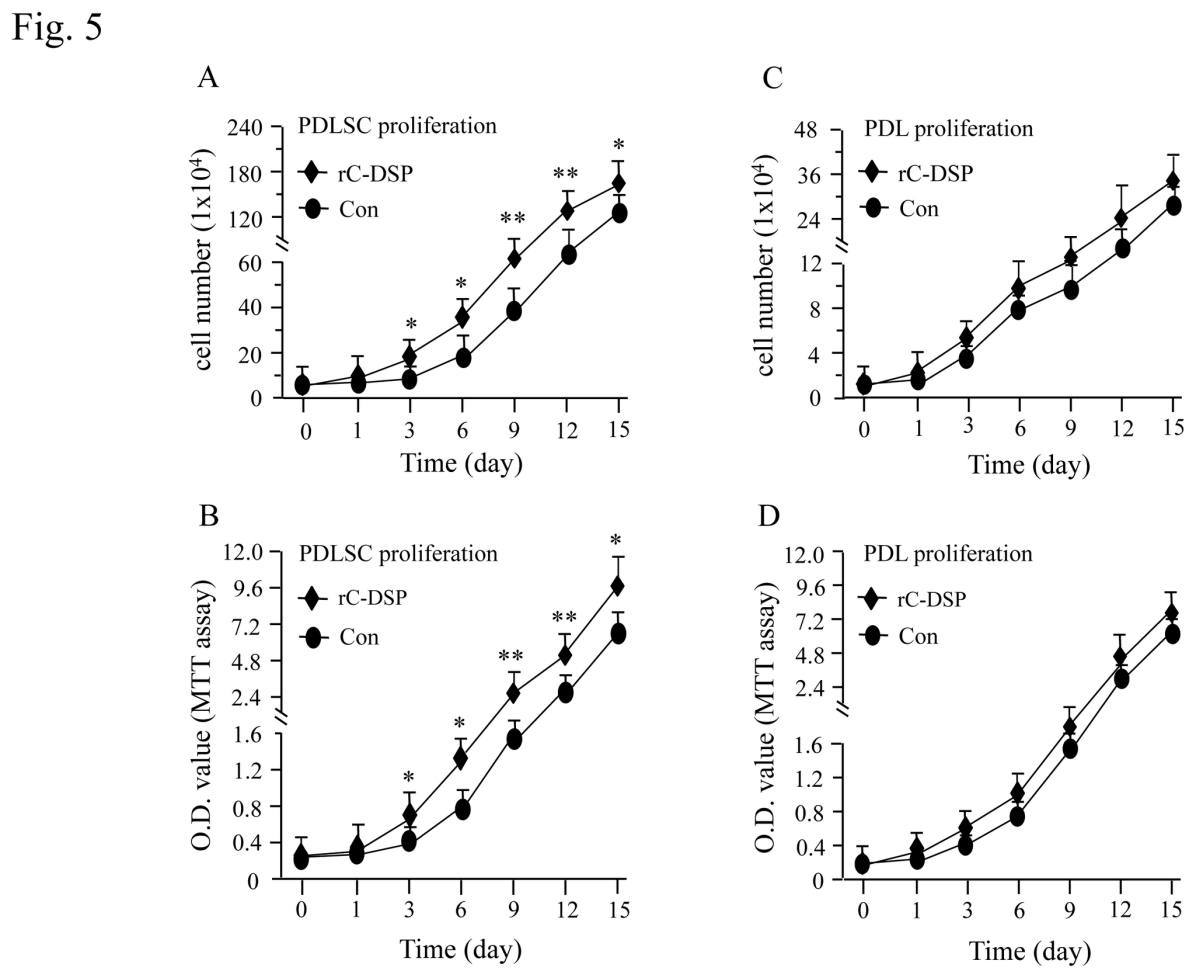
Effect of rC-DSP on cell proliferation. PDLSC and PDL cells were treated with or without 50 mM of rC-DSP at days 3, 6, 9, 12 and 15. Proliferation rate of PDLSC and PDL cells were measured using cell counting and MTT assays. rC-DSP induces PDLSC cell proliferation at days 3, 6, 9, 12 and 15 (**A-B**). There are not different PDL cell proliferation between rC-DSP treated and control groups (**C**-**D**). *p<0.05; **p<0.01. Data are the mean ± S.D. (n = 3) from three independent experiments. Con, control.

**Figure 6:**
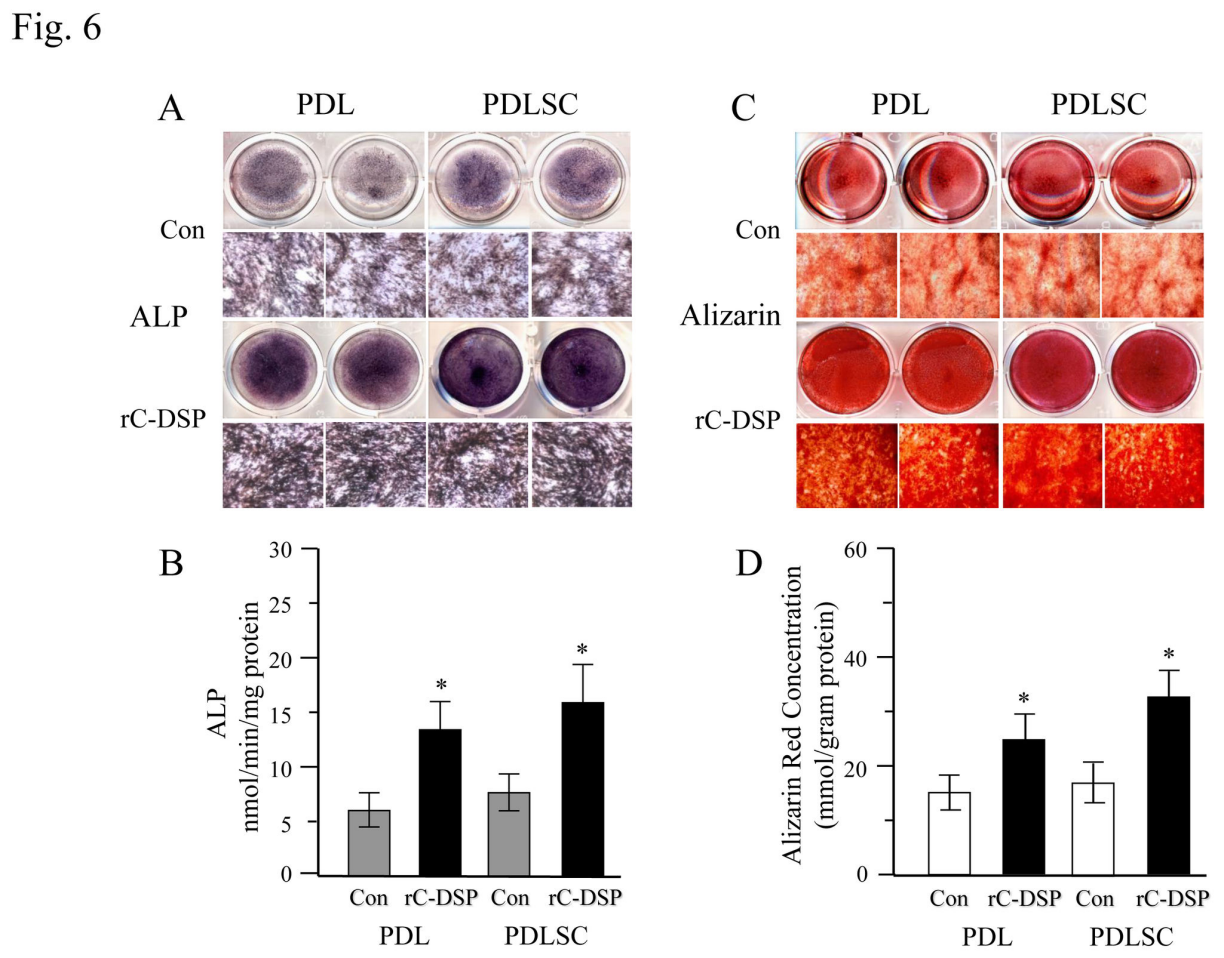
Effect of rC-DSP on cell differentiation and biomineralization. For differentiation assay, the cells were treated with rC-DSP or without rC-DSP (con) for 7 days, ALP activity was determined as described under “Materials and Methods”. (**A**). The cells were fixed and stained for ALP activity. (**B**). Data were means ± S.D. (n=3) from three independent experiments. There were significantly different between rC-DSP treated groups and rC-DSP untreated groups (*p<0.05). (**C**). For cell biomineralization, the cells were maintained in the same condition for 21 days. PDL and PDLSC cells were fixed and stained for Alizarin S Red. (**D**). Calcium deposits were determined by Osteogenesis Assay kit described in “Materials and Methods”. Data represent mean ± S.D. in triplicate from three independent experiments. Con, control.

To assess cell proliferation, the PDL and PDLSC cells were grown in the presence or absence of rC-DSP from 1 day to 15 days. The cell proliferation was calculated using the cell counting and MTT assays. The results showed that rC-DSP stimulated PDLSC proliferation after day 3 induction until day 15 examined (Figure 5A-B) whereas this peptide had no effect on PDL proliferation (Figure 5C-D). However, rC-DSP enhanced GF cell proliferation until day 6 induction (Figure S2A-B). We then studied whether this protein had an inducible effect on the cell differentiation. The cells were treated with the peptide in presence of ascorbated acid and β-glycerophosphate for given time periods. At day 7, ALPase activity, a maker of cell differentiation, was significantly greater in rC-DSP treated cells than the control groups (Figure 6A-B). At 21 days, mineralization deposit in rC-DSP treated PDLSC and PDL cells was higher than that of the control groups by quantitative measurement of the mineralization deposit (Figure 6C-D).

**Figure 7:**
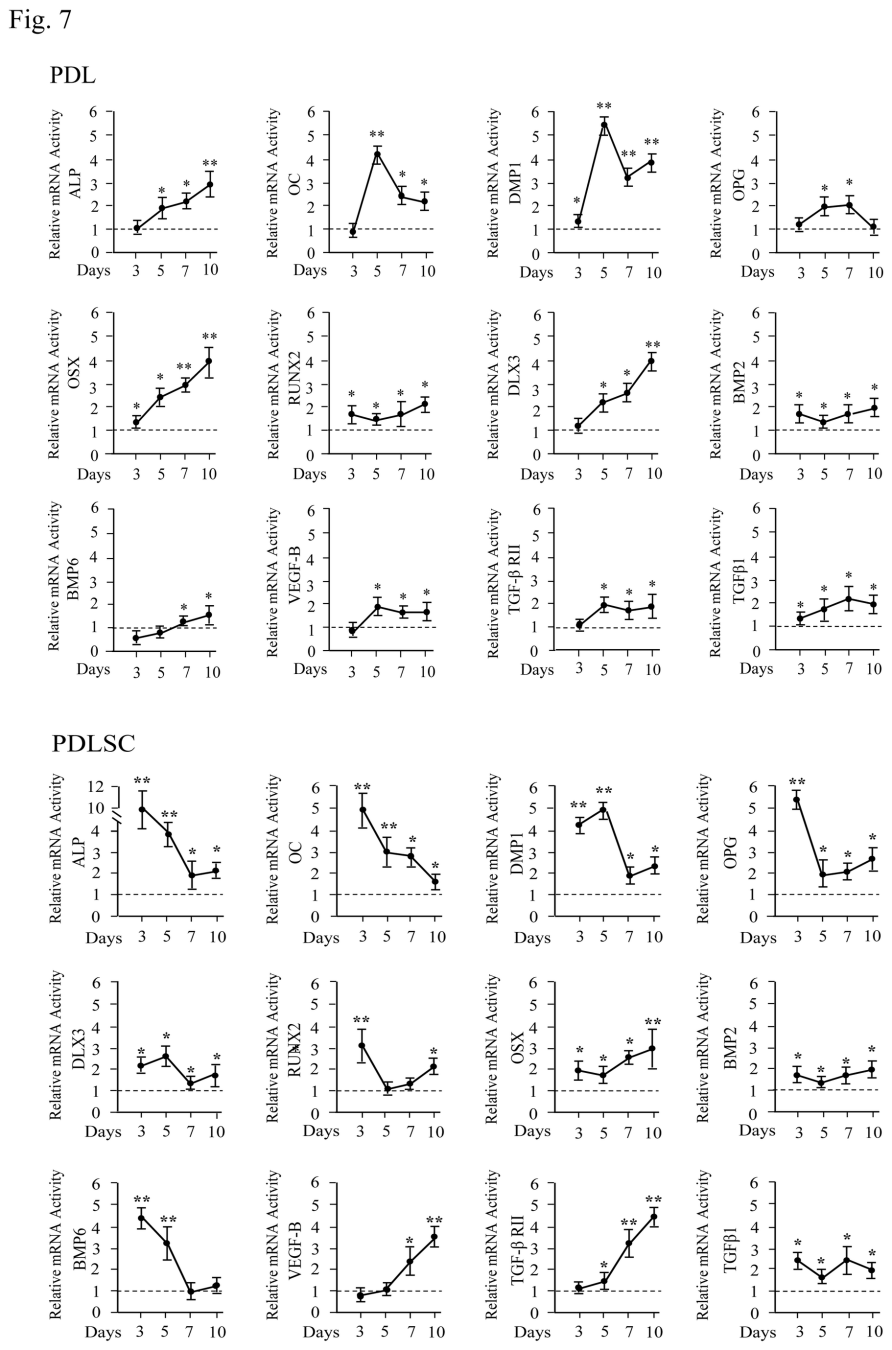
Effect of rC-DSP on the gene expression levels in PDL and PDLSC cells. The cells were treated with or without 50 mM of rC-DSP at 3, 5, 7 and 10 days. The mRNA levels of these genes were analyzed by quantitative RT-PCR. Cyclophilin A was used as an internal control. Expression of those mRNAs in the cells without rC-DSP treatment acts as a 1.0-fold increase. Dotted lines represent control level. The bar graphs show means ± S.D. (n=3) from three independent experiments. Asterisks show significant differences between rC-DSP treated and control cells (*p< 0.05, **p<0.01).

**Figure 8:**
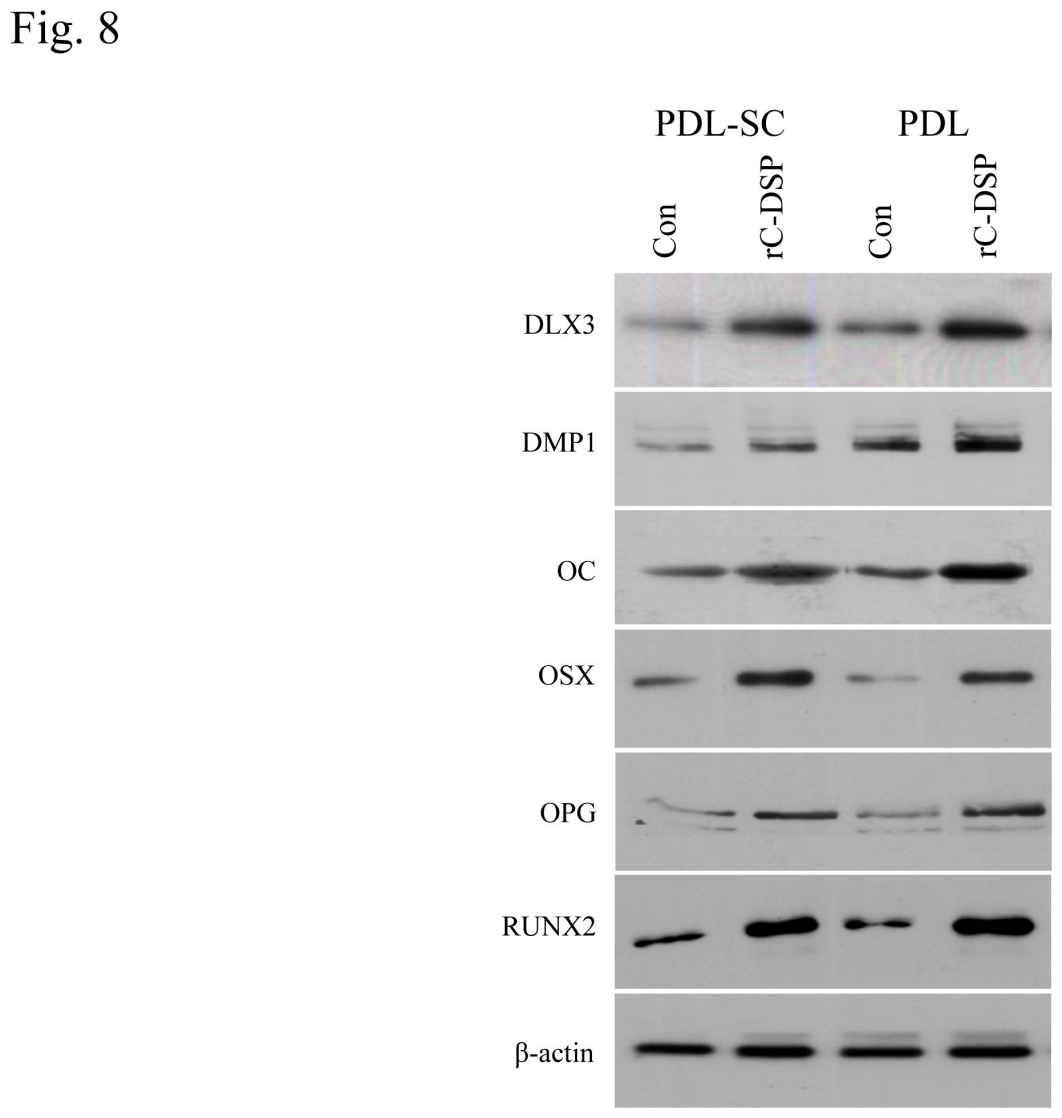
Effect of rC-DSP on protein expression levels in PDL and PDLSC cells. The cells were treated with or without rC-DSP at 7 days. The cells were lysed and protein expressional levels were detected by Western blot assay using the above protein antibodies, respectively. Fifty µg of total cellular lysates were run on 7% SDS-PAGE gels and subsequently electro-blotted. Membranes were blocked with 5% non-fat milk in TBST buffer for 60 min at room temperature and then probed with primary antibodies of a dilution (1:500-1,000) with 1% bovine serum albumin in TBST buffer at 4°C for overnight. After washing, the membranes were incubated with secondary antibodies of a dilution (1:5,000-10,000). Immunoreactivity was determined using ECL chemiluminescence reagent. β-actin was used as an internal control.

Next, we investigated which genes were induced by rC-DSP in these cells. Eighteen gene expressions related to bone and teeth were measured using qRT-PCR. Expression levels of 12 genes among 18 were significantly increased in PDLSC, PDL and GF cells by rC-DSP compared to the control groups (Fig. 7, Fig. S3). This included tooth/bone ECM genes; ALP, DMP1, OC, OPG and transcriptional factors; DLX3, OSX and RUNX2 as well as growth factors and their receptors; BMP2, BMP6, TGF-β1, TGF-βR2 and VEGF-B. Changes of these gene expression levels induced by rC-DSP were dependent on these cell types. Also, we found that several tooth/bone relate gene expressions showed no significant differences between rC-DSP-treated and -untreated groups. It includes BMP1, BMP7, collagen alpha 1, DSPP, LIF and tuftelin. Using Western blot analysis, we further verified effect of rC-DSP on these gene expressions at protein levels (Figure 8, Figure S4).

## Discussion

Periodontal development and regeneration are involved in several types of cells including PDL cells, cementoblasts, gingival fibroblasts and alveolar osteoblasts. Differentiation of these cells from dental follicle is initially derived from mesenchymal neural crest cells [49]. The mesenchymal neural crest cells are multipotent migratory cells capable of self-renewing decision. Differentiation of the mesenchymal neural crest cells into cell-specific fate is controlled by multiple factors including transcription factors, growth factors and extracellular matrix (ECM) although the exact mechanisms of how neural crest cells terminally differentiate into individual lineages or specific target tissues remain to be defined. However, the niche is the *in vivo* microenvironment that is capable of regulating progenitor/stem cell survival, self-renewal, and differentiation. ECM, one of niche components, is involved in growth factor release, cell-cell contact, and cell-matrix adhesion as well as it influences cell behavior and fate [20,21,24]. 

In this study, we investigated effect of rC-DSP on attachment, migration, proliferation and differentiation of PDL and PDLSC cells. We firstly studied expression pattern and distribution of DSP fragments in mouse periodontium at the transcriptional and translational levels using *in situ* hybridization and immunohistochemical analyses. With these approaches, *in situ* hybridization assay documented that DSP expression was observed in PDL and alveolar bone at postnatal day 10 and the later stages of developing mouse teeth (Figure 1).These data suggest that DSP is synthesized and secreted by osteoblasts, PDL cells and gingival fibroblasts that are associated with the initial formation of the periodontium. Previously, our and other groups identified that DSPP is processed into two major dentin extracellular matrix proteins, DSP and DPP [40,42,43], and DSP is furthermore processed into several low molecular weight fragments [41,44]. Using the NH_2_- and COOH-terminal DSP antibodies, we found that expression patterns and levels of the NH_2_- and COOH-terminal DSP domains are some different. The COOH-terminal DSP is expressed in mineralized tissues; highly in dentin and moderately in cementum although its expression is observed in odontoblasts, PDL cells, gingival fibroblasts and osteoblasts. In contrast, expression of the NH_2_-terminal DSP domain is apparently seen in predentin, odontoblasts, dental pulp cells and PDL cells, and weakly in dentin as well as barely in cementum (Figure 2). Previous study indicated that the COOH terminal domain of DSP binds to integrin β6 and activates Smad^1/5/8^ and p38 protein phosphorylation in mouse dental cells [unpublished data]. It suggests that the COOH-terminal DSP fragment(s) may be involved in the maintenance of the periodontal tissue microenvironment.

We then generated the recombinant COOH-terminal DSP peptide (rC-DSP) and coated it on coverslips. We observed that this peptide looks like “fibrils” visible under SEM and is similar to ECM of murine fibroblast (MC3T3-E1) cells [25]. Evans et al. found that the ECM from MC3T3-E1 cells is able to induce murine embryonic stem cell attachment, proliferation and differentiation, promoting osteogenesis [25]. To test whether rC-DSP is mimic like ECM, we cultured PDLSC and PDL cells on rC-DSP coated plates and found that this peptide was able to induce those cell attachment. We assumed that mechanisms of cell attachment induced by rC-DSP are involved in binding of rC-DSP to cellular membrane proteins such as integrin β6. We further observed that rC-DSP promotes cell migration at dose-dependent manner. Sliva et al. reported that when demineralized dentin crude extracts and isolated DSP protein from incisor dentin were injected into the mouse peritoneal cavity, respectively, both the demineralized dentin crude extracts and DSP protein induced neutrophil cell migration into peritoneal cavity, and expression of IL-1β, TNF-α and other chemokines were significantly increased [50]. They suggested that the dentin crude extracts and DSP stimulated neutrophil migration through the synthesis of those chemokines. However, the mechanisms of rC-DSP in PDLSC and PDL cell migration need to be further clarified. For cytotoxic assay, these cells were cultured in different concentrations of rC-DSP at 1, 3, 7 days and cytotoxic activity was measured by LDH. We observed that there no cytotoxic effect on these cells for up to 50 mM of rC-DSP treatment at 7-day culture compared to the control groups. This result indicates that rC-DSP is a safe biomaterial. We then observed that rC-DSP was able to stimulate PDLSC and GF cell proliferation, but not PDL cells. Yun et al. reported that a full-length recombinant DSP enhanced human dental pulp cell proliferation [51]. Therefore, it is likely that effect of rC-DSP on cell proliferation is dependent on cell types. During periodontal regeneration, periodontal ligament cells migrate and attach between alveolar bone and cementum. The enhancement of cell attachment, migration and proliferation by rC-DSP suggests that this peptide is mimic ECM and plays its potential role in periodontal regeneration. 

Besides cell attachment, migration and proliferation, we also noted that rC-DSP promoted PDLSC and PDL cell differentiation and mineralization. The mechanisms of rC-DSP in PDLSC and PDL cell differentiation and mineralization are controlled by multiple bone/tooth-relate genes including OC, DMP1, OPG; and transcription factors; DLX3, OSX and RUNX2 as well as growth factors; BMP2, BMP6, TGFβ1, TGFβ-RII and VEGF-B (Figures 7 and 8). Previous studies demonstrated that differentiation of adipose-derived stromal cells and dental pulp cells were regulated by DSPP and DSP [52,53]. Wu et al. found that DSPP overexpression increased expression of ALP, BSP, DMP1, OC, RUNX2, OSX and other genes in adipose-derived stromal cells [52] while Lee et al. reported that DSP induced dental pulp cell differentiation via the BMP/Smad, JNK, ERK and MAPK signaling pathways [53]. We also found that OPG expression was induced by rC-DSP in the three cell lines. The periodontal ligament cells express both RANKL and OPG [54]. In the periodontal region, tooth eruption and root resorption as well as alveolar bone remodeling require bone resorption and induction. OPG, a soluble decoy receptor of RANKL, competes with RANK for RANKL binding and inhibits osteoclastogenesis and tooth root resorption [55]. Our finding suggests that rC-DSP may confer protection to periodontium by controlling balance between osteoblastogenesis and osteoclastognesis through the RANKL signaling pathway.

Bone morphogenetic proteins (BMPs), transforming growth factor beta (TGFβ) and TGFβ receptors (TGFβ-R) belong to TGFβ superfamily essential to the commitment and differentiation of bone and tooth cell lineages [56,57]. For instance, BMP2 is expressed in bone and dental tissues [58]. Thus, BMP2 is involved in specifying the fate of the mesenchymal cells [59-61]. Moreover, TGFβ signaling promotes dental and bone cell proliferation and differentiation by increasing expression of many transcription factors including DLX3, OSX and RUNX2 [62-64]. In this study, we observed that DLX3, OSX and RUNX2 were enhanced by rC-DSP. Both OSX and RUNX2 are important factors known to be necessary for osteoblast and dental cell lineage commitment and the subsequent cell proliferation and differentiation [65-67]. Mutations of either OSX or RUNX2 gene results in abnormal bone and tooth development and formation [68-70]. Recently, Gao et al. have found that in OSX conditional knock-out mice, cementocyte differentiation and mineralization were impaired [71]. Whether rC-DSP regulates cell differentiation and biomineralization via TGFβ signaling needs to be further investigated. 

More interestingly, we found that rC-DSP induced VEGF-B gene expression in PDL and PDLSC cells. VEGF-B is a member of vascular endothelial growth factor family and has the survival effect on not only vascular endothelial cells, but also on pericytes, smooth muscle cells, and vascular stem/progenitor cells [72]. In addition, we observed that time-course expression pattern of VEGF-B is different between PDLSC and PDL cells. In PDLSC cells, VEGF-B expression was gradually induced by rC-DSP from day 3 to day 10 while in PDL cells, the VEGF-B expression is induced until day 3 of rC-DSP treatment and then kept at a 2-fold increase at days 5, 7 and 10 examined. In both the PDLSC and PDL cells, several gene expression profiles at the time-course rC-DSP induction were similar such as DMP1 and BMP2 while there were differences in other genes. The differences of gene expression patterns induced by rC-DSP at time courses may be dependent on different cell types. Unlike the PDL cells, the PDLSC cells have self-renewable characteristic and cell proliferation are induced by rC-DSP. Different gene expression between the PDL and the PDLSC cells induced by rC-DSP could reflect their unique biological functions and cell behaviors. However, molecular mechanisms of the PDL and the PDLSC cells responsible to rC-DSP have not been completely understood.

Taken together, it indicates that rC-DSP is involved in multiple signaling transduction pathways in regulating the PDLSC and PDL proliferation and differentiation.

## Conclusions

In this study, we found that rC-DSP significantly facilitates attachment, migration and proliferation and differentiation of the PDLSC and PDL cells. Effect of rC-DSP on cell proliferation and differentiation is via regulating gene expression of many tooth/bone markers, growth factors and transcription factors. As a natural product exists in periodontal tissue, the domain of DSP may act as a tissue-specific ECM for primary PDL and PDL stem cell proliferation and differentiation during periodontal repair and regeneration. Future experiments are to investigate effect of rC-DSP on cell proliferation, migration and differentiation in *in vivo* studies.

## Supporting Information

Figure S1 **Effect of rC-DSP on gingival fibroblast damage, attachment and migration.** **A**. GF cells were treated with the given doses of rC-DSP for 1, 3 and 7 days. Lactate dehydrogenase (LDH) from the cell supernatant and extracts was quantitated using CytoTox 96® non-radioactive cytotoxicity assay. Data are shown as mean percentage of damage/dead cells obtained by analysis of three separate experiments. Bar graphs represent means ± S.D. (n=3). There were no significant differences among control and rC-DSP treated groups. Super, cell supernatant. **B**. GF cells were grown on coated with or without different concentrations of rC-DSP plates. After 12 h, adherent cells were fixed and washed with PBS. Crystal violet was then added to the cells and the absorbance of crystal violet taken by cells was measured at 550 nm on an absorbance spectrometer. The results showed that rC-DSP induces GF cell attachments. The study was carried out in three wells from three independent experiments. *p<0.05; **p<0.01. **C**. GF cells were treated with or without different concentrations of rC-DSP for 16 h. Photographs represent the migrated cells. Concentration-dependent effect of rC-DSP was observed in GF cells. Bar represents the mean ± S.D (n=3) from three independent experiments.
(TIF)

Figure S2 **Effect of rC-DSP on cell proliferation.** GF cells were treated with or without 50 mM of rC-DSP at days 3, 6, 9, 12 and 15. Proliferation rate of GF cells were measured using cell counting and MTT assays. rC-DSP induces GF cell proliferation at 6-day treatment until 15 days examined Data are the mean ± S.D. (n=3) from three independent experiments. *p <0.05; **p <0.01. Con, control.
(TIF)

Figure S3 **Effect of rC-DSP on the gene expression levels in GF cells.** The cells were treated with or without 50 mM of rC-DSP at 3, 5, 7 and 10 days. The mRNA levels of these genes were analyzed by quantitative RT-PCR. Cyclophilin A was used as an internal control. Expression of those mRNAs in the cells without rC-DSP treatment acts as a 1.0-fold increase. Dotted lines represent control level. Similar results were obtained in triplicate of three independent experiments. Asterisks show significant differences between rC-DSP treated and control cells (* *p* < 0.05, ** *p* <0.01).
(TIF)

Figure S4 **Effect of rC-DSP on protein expression levels in GF cells.** The cells were treated with or without rC-DSP at 7 days. The cells were lysed with RIPA buffer and fifty µg of total cellular lysates were run on 7% SDS-PAGE gels. The gels were transferred to Trans-Blot membranes and the membranes were blocked as well as probed with primary antibodies against the above proteins, respectively. After washing, the membranes were incubated with secondary antibodies of a dilution (1:5,000-10,000). Immunoreactivity was determined using ECL chemiluminescence reagent. β-actin was used as an internal control.
(TIF)

Table S1 **Primers used for qRT-PCR.**
(PPTX)

Table S2 **Primers used for qRT-PCR.**
(PPTX)
