# Supplementary figures and images for: Domain of Dentine Sialoprotein Mediates Proliferation and Differentiation of Human Periodontal Ligament Stem Cells
Source: PLoS One. 2013 Dec 3;8(12):e81655. doi: 10.1371/journal.pone.0081655 (PMC3882282; doi:10.1371/journal.pone.0081655)

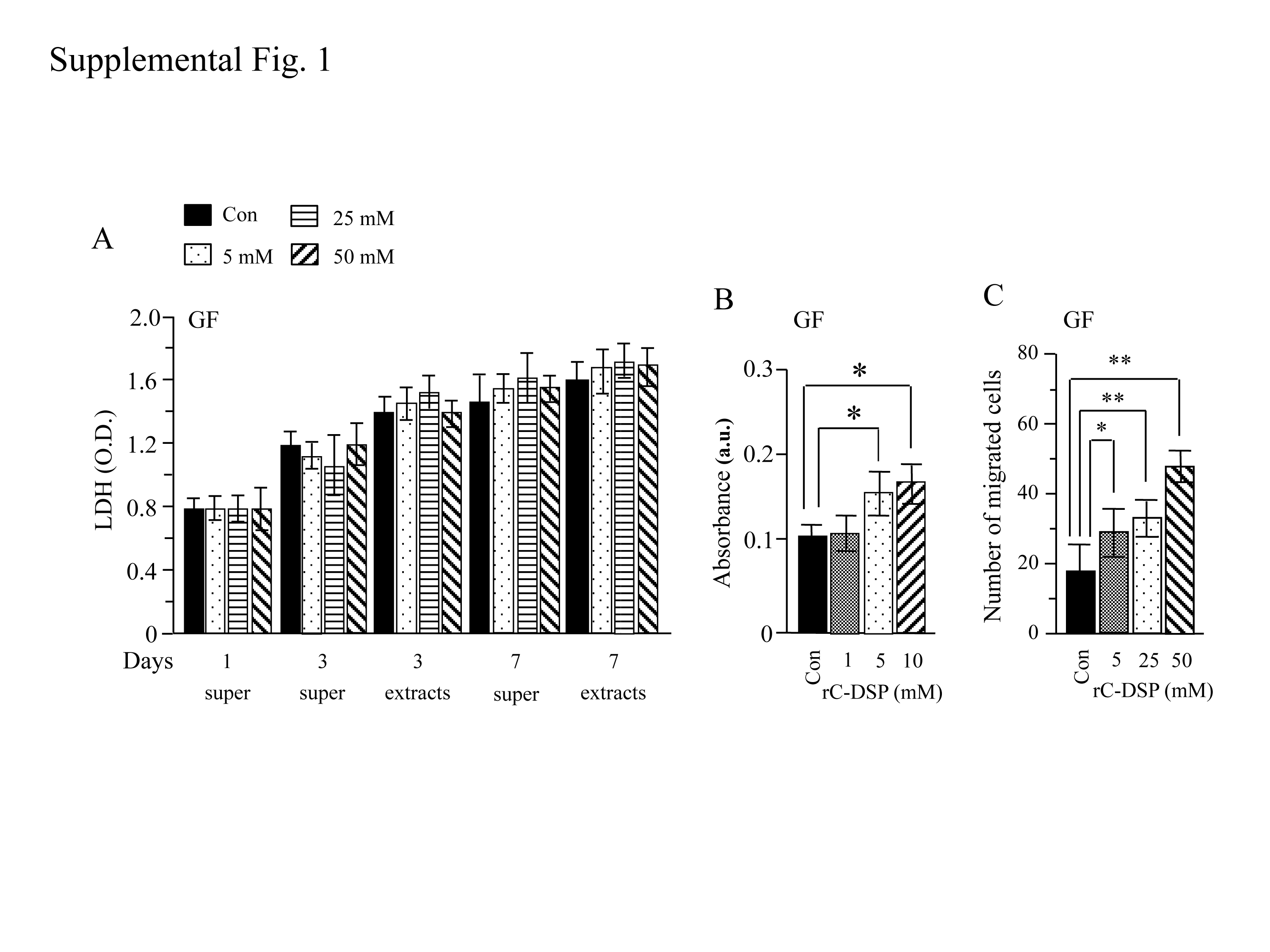

Supplement: Figure S1 — Effect of rC-DSP on gingival fibroblast damage, attachment and migration. A. GF cells were treated with the given doses of rC-DSP for 1, 3 and 7 days. Lactate dehydrogenase (LDH) from the cell supernatant and extracts was quantitated using CytoTox 96® non-radioactive cytotoxicity assay. Data are shown as mean percentage of damage/dead cells obtained by analysis of three separate experiments. Bar graphs represent means ± S.D. (n=3). There were no significant differences among control and rC-DSP treated groups. Super, cell supernatant. B. GF cells were grown on coated with or without different concentrations of rC-DSP plates. After 12 h, adherent cells were fixed and washed with PBS. Crystal violet was then added to the cells and the absorbance of crystal violet taken by cells was measured at 550 nm on an absorbance spectrometer. The results showed that rC-DSP induces GF cell attachments. The study was carried out in three wells from three independent experiments. *p<0.05; **p<0.01. C. GF cells were treated with or without different concentrations of rC-DSP for 16 h. Photographs represent the migrated cells. Concentration-dependent effect of rC-DSP was observed in GF cells. Bar represents the mean ± S.D (n=3) from three independent experiments. (TIF) [file pone.0081655.s001.tif]

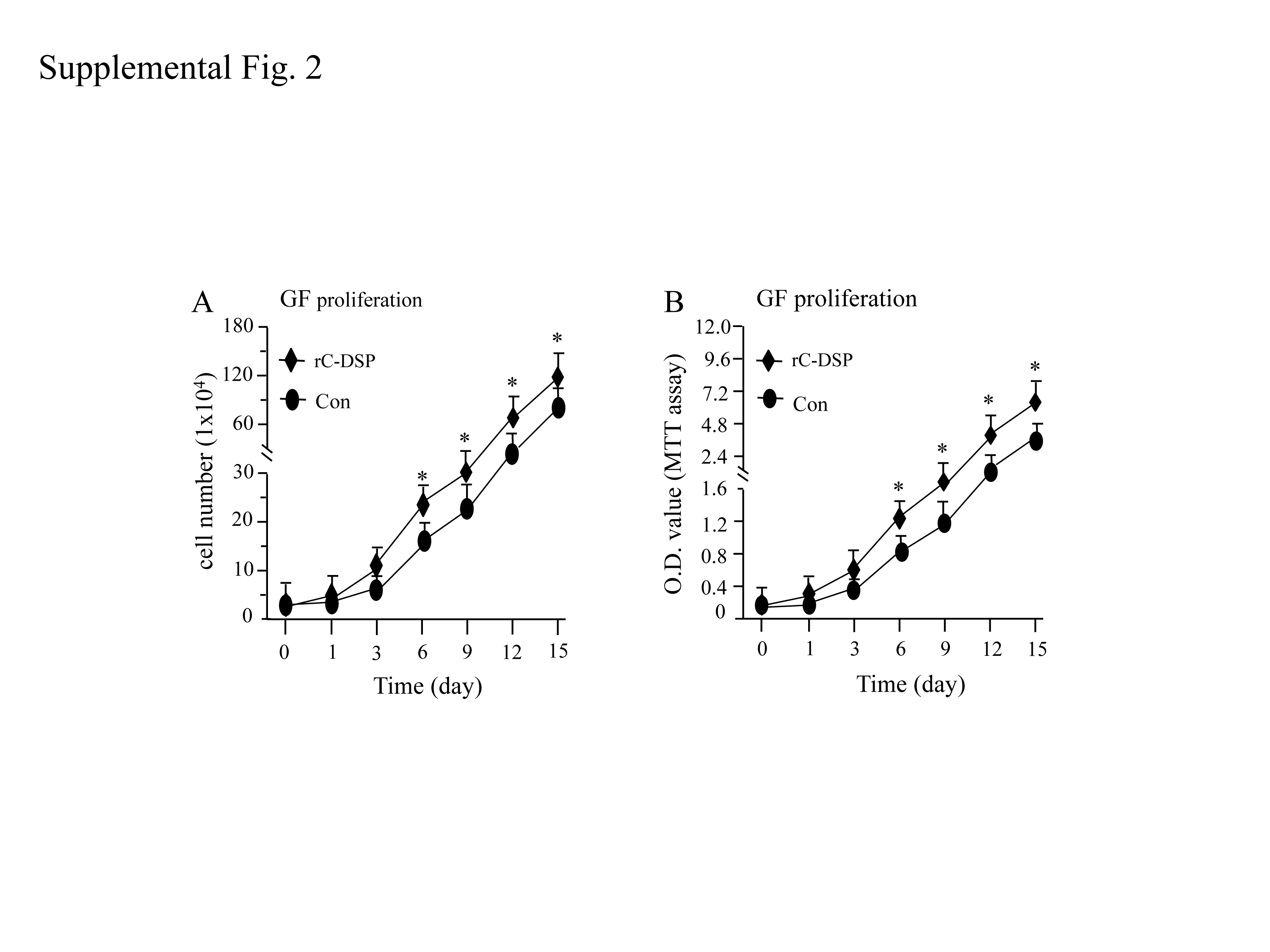

Supplement: Figure S2 — Effect of rC-DSP on cell proliferation. GF cells were treated with or without 50 mM of rC-DSP at days 3, 6, 9, 12 and 15. Proliferation rate of GF cells were measured using cell counting and MTT assays. rC-DSP induces GF cell proliferation at 6-day treatment until 15 days examined Data are the mean ± S.D. (n=3) from three independent experiments. *p <0.05; **p <0.01. Con, control. (TIF) [file pone.0081655.s002.tif]

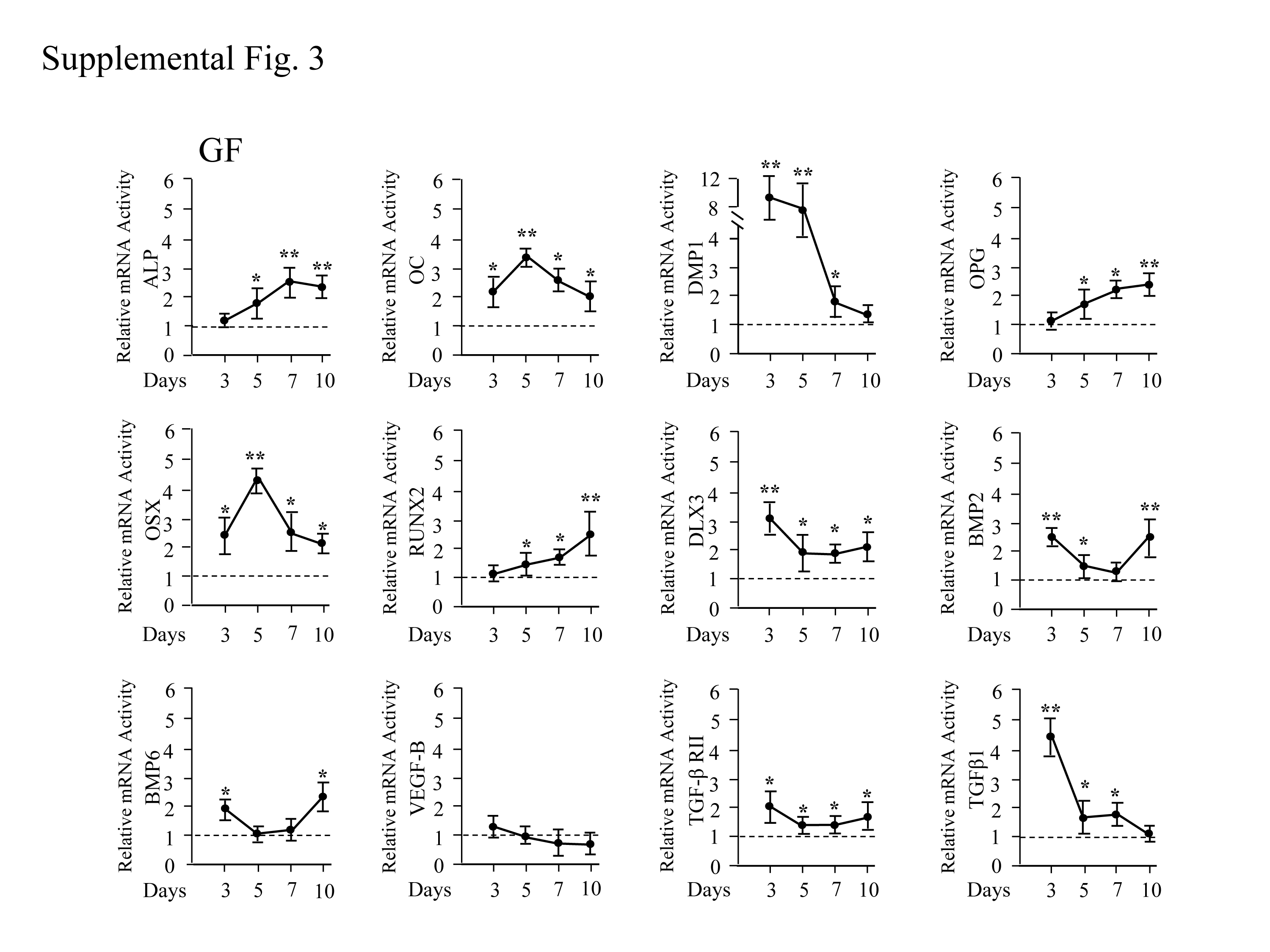

Supplement: Figure S3 — Effect of rC-DSP on the gene expression levels in GF cells. The cells were treated with or without 50 mM of rC-DSP at 3, 5, 7 and 10 days. The mRNA levels of these genes were analyzed by quantitative RT-PCR. Cyclophilin A was used as an internal control. Expression of those mRNAs in the cells without rC-DSP treatment acts as a 1.0-fold increase. Dotted lines represent control level. Similar results were obtained in triplicate of three independent experiments. Asterisks show significant differences between rC-DSP treated and control cells (* p < 0.05, ** p <0.01). (TIF) [file pone.0081655.s003.tif]

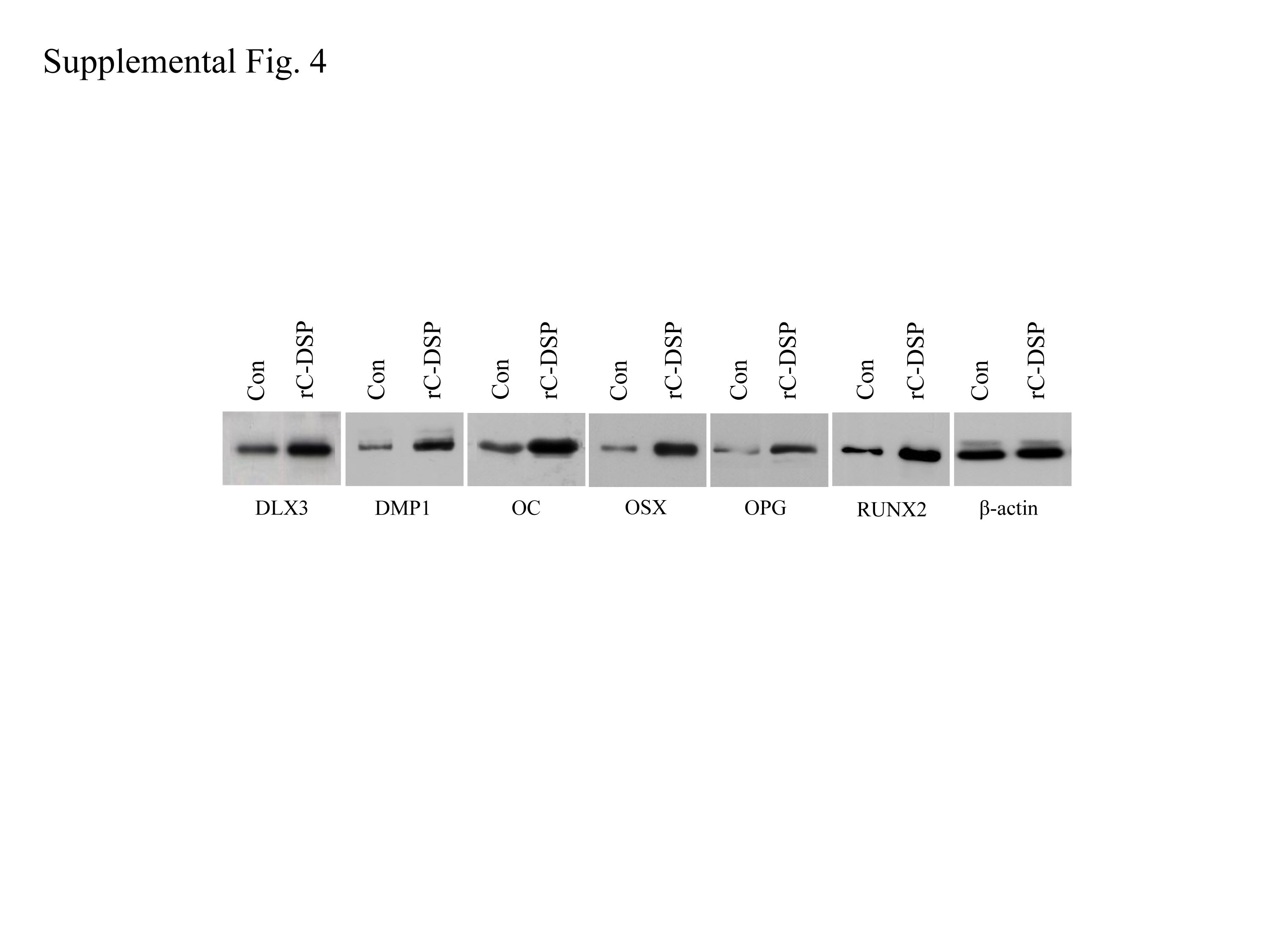

Supplement: Figure S4 — Effect of rC-DSP on protein expression levels in GF cells. The cells were treated with or without rC-DSP at 7 days. The cells were lysed with RIPA buffer and fifty µg of total cellular lysates were run on 7% SDS-PAGE gels. The gels were transferred to Trans-Blot membranes and the membranes were blocked as well as probed with primary antibodies against the above proteins, respectively. After washing, the membranes were incubated with secondary antibodies of a dilution (1:5,000-10,000). Immunoreactivity was determined using ECL chemiluminescence reagent. β-actin was used as an internal control. (TIF) [file pone.0081655.s004.tif]
