# Supplementary material for: Domain of Dentine Sialoprotein Mediates Proliferation and Differentiation of Human Periodontal Ligament Stem Cells
Source: PLoS One. 2013 Dec 3;8(12):e81655. doi: 10.1371/journal.pone.0081655 (PMC3882282; doi:10.1371/journal.pone.0081655)
Supplement: Table S1 — Primers used for qRT-PCR. (PPTX) [file pone.0081655.s005.pptx]

## Slide 1
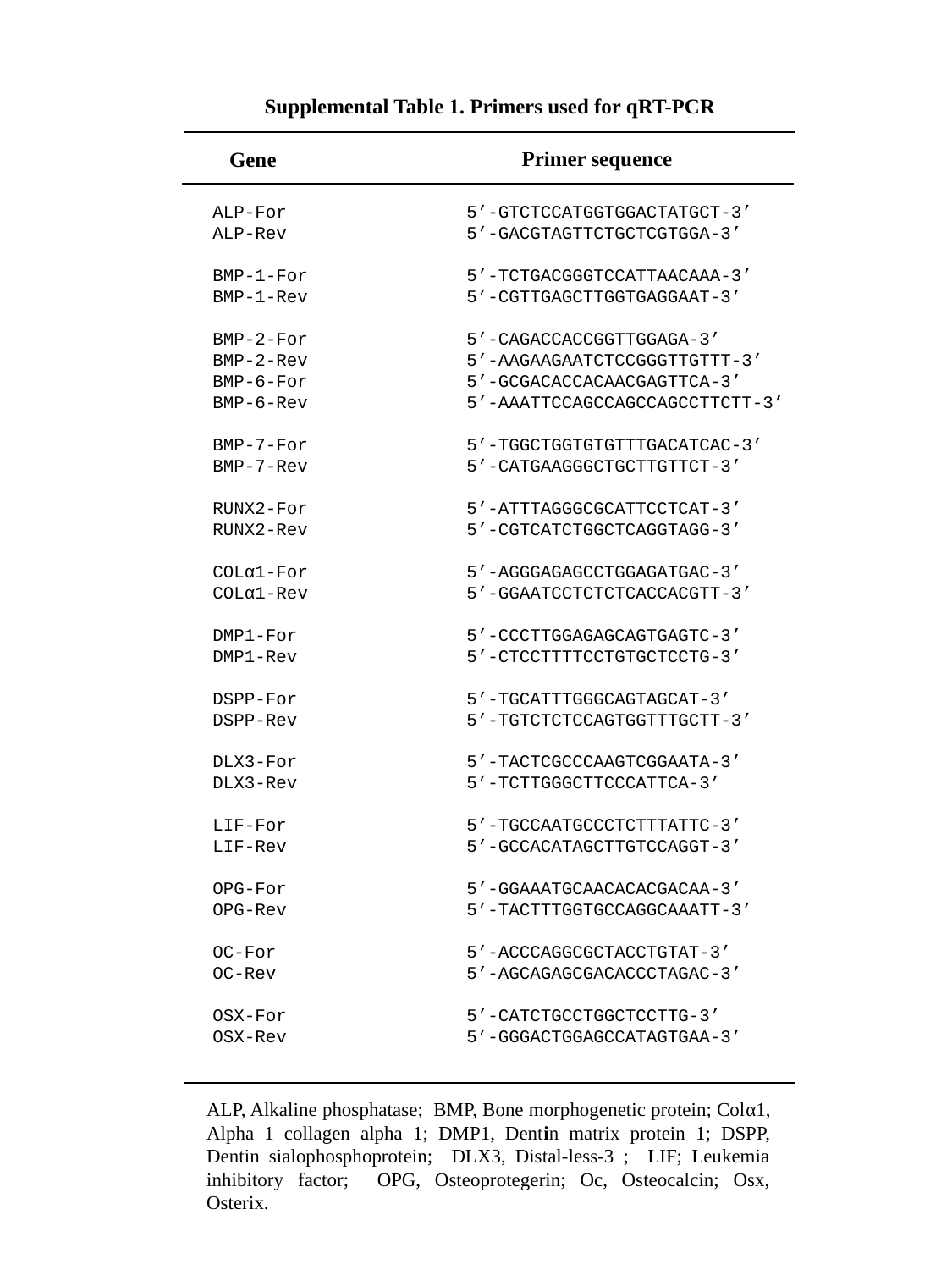

Supplemental Table 1. Primers used for qRT-PCR
Primer sequence
Gene
ALP-For		5’-GTCTCCATGGTGGACTATGCT-3’
ALP-Rev		5’-GACGTAGTTCTGCTCGTGGA-3’
BMP-1-For		5’-TCTGACGGGTCCATTAACAAA-3’
BMP-1-Rev		5’-CGTTGAGCTTGGTGAGGAAT-3’
BMP-2-For		5’-CAGACCACCGGTTGGAGA-3’
BMP-2-Rev		5’-AAGAAGAATCTCCGGGTTGTTT-3’
BMP-6-For 		5’-GCGACACCACAACGAGTTCA-3’
BMP-6-Rev 		5’-AAATTCCAGCCAGCCAGCCTTCTT-3’
BMP-7-For 		5’-TGGCTGGTGTGTTTGACATCAC-3’
BMP-7-Rev 		5’-CATGAAGGGCTGCTTGTTCT-3’
RUNX2-For		5’-ATTTAGGGCGCATTCCTCAT-3’
RUNX2-Rev		5’-CGTCATCTGGCTCAGGTAGG-3’
COLα1-For 		5’-AGGGAGAGCCTGGAGATGAC-3’
COLα1-Rev 		5’-GGAATCCTCTCTCACCACGTT-3’
DMP1-For		5’-CCCTTGGAGAGCAGTGAGTC-3’
DMP1-Rev		5’-CTCCTTTTCCTGTGCTCCTG-3’
DSPP-For		5’-TGCATTTGGGCAGTAGCAT-3’
DSPP-Rev		5’-TGTCTCTCCAGTGGTTTGCTT-3’
DLX3-For		5’-TACTCGCCCAAGTCGGAATA-3’
DLX3-Rev		5’-TCTTGGGCTTCCCATTCA-3’
LIF-For		5’-TGCCAATGCCCTCTTTATTC-3’
LIF-Rev		5’-GCCACATAGCTTGTCCAGGT-3’
OPG-For 		5’-GGAAATGCAACACACGACAA-3’
OPG-Rev 	 5’-TACTTTGGTGCCAGGCAAATT-3’
OC-For 		5’-ACCCAGGCGCTACCTGTAT-3’
OC-Rev 		5’-AGCAGAGCGACACCCTAGAC-3’
OSX-For		5’-CATCTGCCTGGCTCCTTG-3’
OSX-Rev		5’-GGGACTGGAGCCATAGTGAA-3’
ALP, Alkaline phosphatase; BMP, Bone morphogenetic protein; Colα1, Alpha 1 collagen alpha 1; DMP1, Dentin matrix protein 1; DSPP, Dentin sialophosphoprotein; DLX3, Distal-less-3 ; LIF; Leukemia inhibitory factor; OPG, Osteoprotegerin; Oc, Osteocalcin; Osx, Osterix.
