# Supplementary material for: Domain of Dentine Sialoprotein Mediates Proliferation and Differentiation of Human Periodontal Ligament Stem Cells
Source: PLoS One. 2013 Dec 3;8(12):e81655. doi: 10.1371/journal.pone.0081655 (PMC3882282; doi:10.1371/journal.pone.0081655)
Supplement: Table S2 — Primers used for qRT-PCR. (PPTX) [file pone.0081655.s006.pptx]

## Slide 1
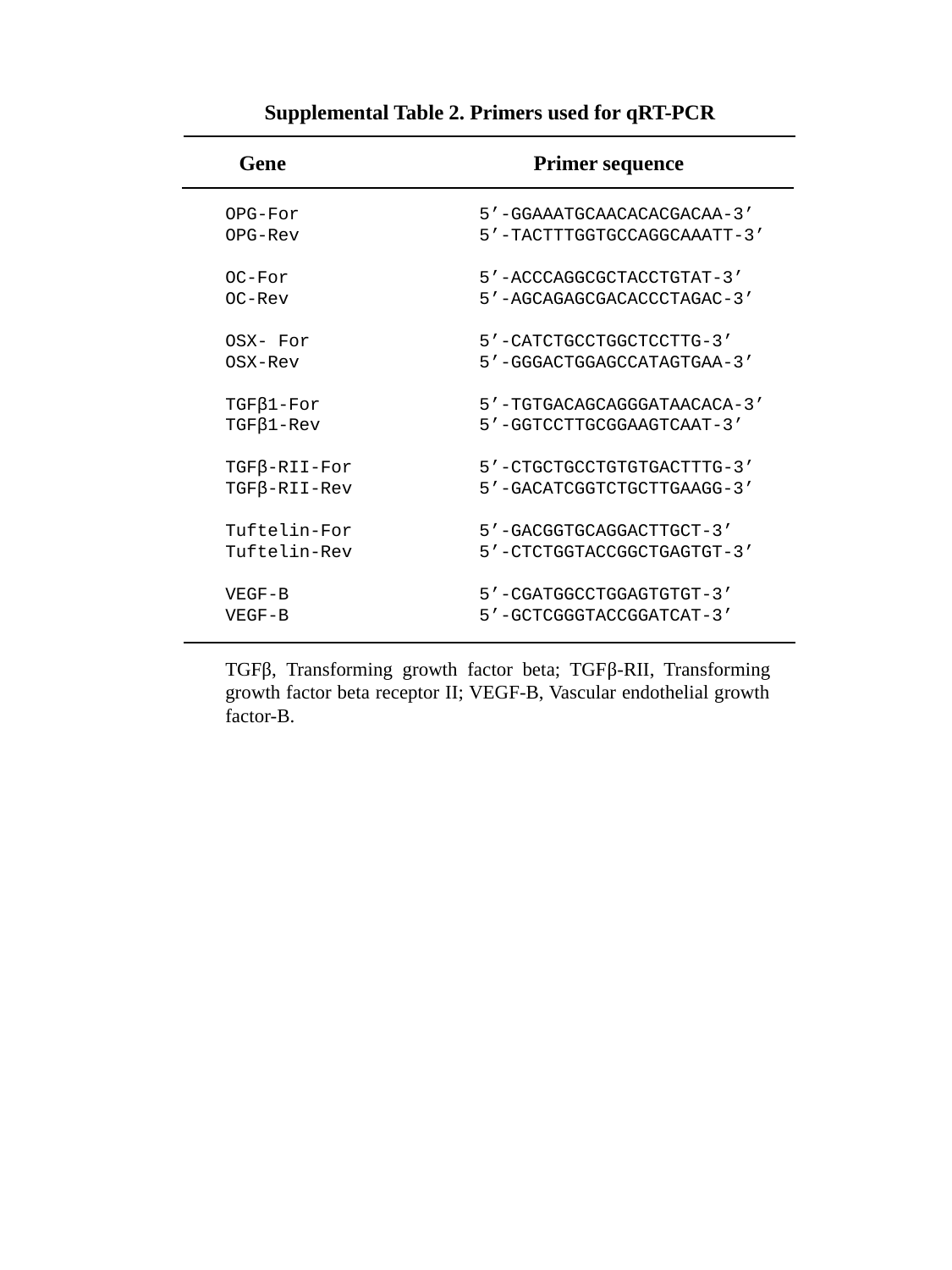

Supplemental Table 2. Primers used for qRT-PCR
Gene
Primer sequence
OPG-For 		5’-GGAAATGCAACACACGACAA-3’
OPG-Rev 	 5’-TACTTTGGTGCCAGGCAAATT-3’
OC-For 		5’-ACCCAGGCGCTACCTGTAT-3’
OC-Rev 		5’-AGCAGAGCGACACCCTAGAC-3’
OSX- For		5’-CATCTGCCTGGCTCCTTG-3’
OSX-Rev		5’-GGGACTGGAGCCATAGTGAA-3’
TGFβ1-For 		5’-TGTGACAGCAGGGATAACACA-3’
TGFβ1-Rev		5’-GGTCCTTGCGGAAGTCAAT-3’
TGFβ-RII-For 5’-CTGCTGCCTGTGTGACTTTG-3’
TGFβ-RII-Rev	5’-GACATCGGTCTGCTTGAAGG-3’
Tuftelin-For	5’-GACGGTGCAGGACTTGCT-3’
Tuftelin-Rev	5’-CTCTGGTACCGGCTGAGTGT-3’
VEGF-B		5’-CGATGGCCTGGAGTGTGT-3’
VEGF-B		5’-GCTCGGGTACCGGATCAT-3’
TGFβ, Transforming growth factor beta; TGFβ-RII, Transforming growth factor beta receptor II; VEGF-B, Vascular endothelial growth factor-B.
